# Establishment of methods for visual and rapid detection of piscine lactococcosis based on isothermal recombinase polymerase amplification

**DOI:** 10.1186/s12917-026-05587-5

**Published:** 2026-05-28

**Authors:** Hanyu Luo, Zhihao Wu, Weiqi Fan, Hui Zeng, Jie Li, Yong-An Zhang, Yang Zhou

**Affiliations:** 1 National Key Laboratory of Agricultural Microbiology, Hubei Hongshan Laboratory, Hainan Research Institute, College of Biomedicine and Health, College of Fisheries, Huazhong Agricultural University, Wuhan, China; 2 Hubei Jiangxia Laboratory, Wuhan, China; 3 Key Laboratory of Aquaculture Facilities Engineering, Ministry of Agriculture and Rural Affairs, Wuhan, China

**Keywords:** Lactococcosis, Recombinase polymerase amplification (RPA), Lateral flow dipstick (LFD), Rapid detection, *Lactococcus* species

## Abstract

**Background:**

Lactococcosis is a bacterial disease in fish caused by several *Lactococcus* species, including *L. garvieae* and *L. petauri*, with clinical signs frequently overlapping with those caused by other streptococcal species. This diagnostic overlap necessitates a prompt and precise diagnostic tool for guiding accurate treatment and control. To address this diagnostic challenge, we developed basic recombinase polymerase amplification (basic-RPA) and RPA combined with lateral flow dipstick (RPA-LFD) assays for the detection of lactococcosis*-*associated *Lactococcus* species.

**Results:**

Both methods exhibited a detection limit of 10^3^ copies/µL for the recombinant plasmid pMD19-*adhE*-*ywdF* and 10 pg/µL for *L. garvieae* genomic DNA. Specificity testing using DNA from *L. garvieae*, *Streptococcus agalactiae*, *Aeromonas hydrophila*, *Edwardsiella piscicida*, and *Streptococcus iniae* confirmed that amplification occurred exclusively with *L. garvieae*, showing specificity against the tested panel. The assay also detected *L. petauri*, indicating that it recognizes both major lactococcosis-associated *Lactococcus* species. When tested with the plasmid standard, the RPA assays were 100-fold more sensitive than PCR; with genomic DNA, all three methods showed the same detection limit. When applied to 20 samples from experimentally infected tilapia, the RPA-LFD results were fully consistent with those of conventional PCR.

**Conclusions:**

The established methods are straightforward, sensitive, and specific, offering a promising candidate tool for the rapid on-site diagnosis of piscine lactococcosis.

**Supplementary Information:**

The online version contains supplementary material available at 10.1186/s12917-026-05587-5.

## Background

 Lactococcosis is a bacterial disease that usually occurs in fish in the form of meningoencephalitis and acute haemorrhagic sepsis, with an incidence and mortality rate of between 20% and 50% [1]. Lactococcosis is caused by *Lactococcus garvieae*, *L. petauri*, and *L. formosensis*, with *L. garvieae* being the main pathogen [2–4]. *L. garvieae*, a Gram-positive, facultatively anaerobic bacterium, belongs to the genus *Lactococcus* within the family Streptococcaceae. It has been reported that *L. garvieae* begins to proliferate at water temperatures around 18 °C, with its pathogenicity increasing substantially above 20 °C [2, 4]. Notably, this temperature range overlaps with the optimal conditions for the outbreak of streptococcosis. Furthermore, the clinical manifestations of *L. garvieae* infection closely resemble those caused by *Streptococcus* spp., commonly including corneal opacity, abdominal distension, and hemorrhages in visceral organs and skin. Histopathological examination further reveals characteristic lesions, including meningitis and septicemia, in the affected fish [5]. This pathogen has a broad host range and has been identified in multiple fish hosts, including tuna (*Thunnus* spp.), red sea bream (*Pagrus major*), rainbow trout (*Oncorhynchus mykiss*), Japanese eel (*Anguilla japonica*), and Nile tilapia (*Oreochromis niloticus*) [2]. The primary transmission routes of *L. garvieae* are horizontal, through oral, waterborne, and fecal pathways. Diseased fish and asymptomatic carriers serve as the main sources of infection; the pathogen can be shed into water via feces, subsequently infecting healthy fish through the gills, skin wounds, or the oral route [4]. Outbreaks of lactococcosis have inflicted significant economic damage on aquaculture operations.

In recent years, lactococcosis outbreaks have been reported in diverse geographical regions and new host species. In 2023, an outbreak of haemorrhagic septicemia caused by *L. garvieae* occurred in European seabass (*Dicentrarchus labrax*) farms in Spain, associated with water temperatures around 25 °C, resulting in cumulative mortalities of 5–10% [6]. During the same summer, an outbreak in an Italian seabass farm showed lower monthly mortality (2–3%), yet antimicrobial treatments with flumequine and erythromycin proved ineffective [7]. Beyond fish, *L. garvieae* has recently been identified as the etiological agent of white muscle disease in freshwater prawn (*Macrobrachium rosenbergii*) in India [8], and as a pathogen causing mass mortality in cultured pufferfish (*Takifugu obscurus*) in China [9]. Moreover, the first reported outbreak of *L. garvieae* with concurrent *S. agalactiae* infection in farmed red tilapia occurred in Thailand [5]. These developments indicate that *L. garvieae* continues to expand its host range and geographic footprint, posing mounting challenges to global aquaculture.

Accurate and timely diagnosis is crucial for effective control and management of lactococcosis in aquaculture. Although a variety of diagnostic methods for *L. garvieae* have been established, their practical application in field settings remains challenging. Current detection approaches mainly include traditional biochemical identification [10], polymerase chain reaction (PCR) [11], real-time fluorescence quantitative PCR (qPCR) [12], and loop-mediated isothermal amplification (LAMP) [13]. However, PCR and qPCR involve complex procedures and require specialized equipment, limiting their suitability for rapid on-site detection [11, 12]. Additionally, cross-reactivity with related bacteria such as *S. agalactiae* can affect specificity and lead to false-positive results [14, 15]. Furthermore, LAMP typically requires incubation at 60–65 °C [13], which constrains its use in resource-constrained or field settings. Consequently, there exists a pressing need for a simple, rapid, accurate, and field-deployable detection platform to enable early diagnosis of *L. garvieae* infections at the point of need.

Functioning as an innovative isothermal nucleic acid amplification technique, recombinase polymerase amplification (RPA) offers notable advantages including operational simplicity, rapid turnaround, and high specificity under low-temperature conditions, rendering it particularly suitable for point-of-care pathogen detection [16–18]. RPA is typically conducted under consistently low-temperature conditions (37–42℃) and can be completed within 20–30 min. With its requirement for only basic equipment, RPA offers strong potential for field-deployable pathogen detection [19]. The RPA reaction mechanism is driven by three essential protein components: recombinase, a strand-displacing DNA polymerase, and a single-stranded DNA-binding protein (SSB) [20, 21]. Amplification products generated by RPA can be analyzed through multiple detection platforms, including agarose gel electrophoresis, lateral flow dipsticks (LFDs), and real-time fluorescence monitoring. In particular, the RPA-LFD format combines isothermal amplification with immunochromatographic detection, enabling rapid, visual, and equipment-minimal diagnosis in settings such as experimental animal facilities, livestock farms, and clinical point-of-care settings [18, 22, 23].

This study established an RPA-based detection platform for piscine lactococcosis, which includes both a basic RPA assay and an RPA-LFD format. Following the design and screening of specific primers, key reaction parameters such as temperature and time were systematically optimized. The comprehensive assessment of the sensitivity and specificity was conducted for the developed RPA assay. Furthermore, its detection reliability was validated using tissues from experimentally infected tilapia. Ultimately, a detection platform characterized by rapidity, sensitivity, specificity, and operational simplicity was developed, which may offer a practical tool for the timely diagnosis and management of lactococcosis.

## Materials and methods

### Fish and strains

In this study, tilapia averaging 50 ± 10 g in body weight were reared under tank-based aquaculture conditions equipped with a recirculating water system. A consistent water temperature of 26 ± 1 °C was upheld during the study, and the fish were fed a conventional formulated feed twice daily.

*L. garvieae* (strain HZAU2339) and *S. agalactiae* (strain HN016) were originally isolated from diseased tilapia, with the latter described in our previous study [24]. *E. piscicida* (strain HZAU2272), *A. hydrophila* (strain HZAU2273), and *S. iniae* (strain HZAU2275) were isolated from yellow catfish (*Pelteobagrus fulvidraco*) in Hubei Province, China, in 2022. Additionally, *L. petauri* (isolated from *Acipenser baerii*) and *L. lactis* (isolated from *Micropterus salmoides* in 2023) were used as reference strains for specificity testing. All isolates are preserved in the Laboratory of Aquatic Animal Diseases, Huazhong Agricultural University (HZAU collection) and stored in glycerol or milk powder at -80 °C.

### Nucleic acid extraction and generation of standard DNA

All bacterial strains were streaked from − 80 °C glycerol stocks onto THB plates and incubated at 28 °C for 24 h. Subsequently, single colonies were inoculated into 5 mL of THB broth and grown overnight at 28 °C on a constant temperature shaker. Bacterial cells were harvested by centrifugation at 4,000 × *g* for 5 min, and the pellet was used for genomic DNA extraction. Genomic DNA from all bacterial strains was obtained with the HiPure Bacterial DNA Kit (Magen) and preserved at -20℃ before subsequent analysis. To generate a *L. garvieae* DNA standard for RPA assays, a 576 bp fragment encompassing the target region was amplified from *L. garvieae* genomic DNA using primers PCR1-Forward and PCR1-Reverse (Table 1). The amplified PCR fragment was ligated into the pMD19-T vector employing the pMD19-T Vector Cloning Kit (Takara) following the supplier’s protocol. After transforming *Escherichia coli* DH5α competent cells with the ligation product, candidate colonies were selected and subsequently verified via Sanger sequencing employing M13 universal primers. The resulting recombinant plasmid was extracted and purified with the HiPure Plasmid/BAC EF Mini Kit (Magen), and its concentration quantified using a NanoDrop ND-2000c spectrophotometer. The copy number of DNA molecules was calculated by the following formula: amount (copies/µL) = 6.02 × 10^23^ × plasmid concentration (ng/µL) × 10^− 9^ / (number of plasmid bases × 660).

### Design of primers and probes

To ensure high specificity for *L. garvieae*, a novel molecular target was selected through a comparative genomics approach. Complete genome sequences of *L. garvieae* (GenBank: AP027239.1) and the closely related pathogen *S. agalactiae* (GenBank: CP011325.1) were retrieved from the NCBI database. Using TBtools software, a whole-genome comparison was performed to identify genomic regions unique to *L. garvieae*. The specificity of the chosen target sequences was further validated through BLASTn analysis using the GenBank database (https://blast.ncbi.nlm.nih.gov/Blast.cgi).

The RPA assay targets a conserved region spanning the 3′ end of *adhE* to the 5′ end of *ywdF* (designated *adhE-ywdF*). Based on the identified target region, primers and probe were designed in SnapGene software, and their corresponding sequences are provided in Table 1. These oligos, synthesized commercially by Tsingke Biotechnology, were subsequently utilized in RPA assays with *L. garvieae* genomic DNA serving as the template for validation.

### Establishment of basic-RPA and RPA-LFD assays

The basic-RPA was conducted with the DNA Isothermal Rapid Amplification Kit (Basic Type; Amplification Future) in accordance with the manufacturer’s guidelines. The 50 µL reaction mixture comprised 29.4 µL of Buffer A, 2 µL per forward and reverse primer (10 µM), 5 µL of template DNA (1 ng/µL), and 9.1 µL of nuclease-free water. To initiate amplification, 2.5 µL of Buffer B was added to the mixture. Post-amplification, the resulting RPA amplicons were cleaned using Genomic DNA Extraction Phenol Reagent Type I (ChuChengkj) and then subjected to electrophoretic separation on a 2% agarose gel for analysis.

The RPA-LFD assay was carried out with the DNA Isothermal Rapid Amplification Kit (Colloidal Gold Test Strip Type; Amplification Future). Each 50 µL amplification system included 29.4 µL of Buffer A, 2 µL each of forward and reverse primers (10 µM), 0.2 µL of nfo probe (10 µM), 2 µL of template DNA (1 ng/ µL), and 11.9 µL of nuclease-free water. To initiate amplification, 2.5 µL of Buffer B was added to the mixture. Following amplification, a 20-fold dilution of the amplicon was prepared with nuclease‑free water. Subsequently, 80 µL of the diluted amplicon was applied to the sample pad of the lateral flow dipstick (LFD; Amplification Future, China, Cat. No. WLFS8204). Following a 5- min development period, results were interpreted according to the following criteria: the simultaneous appearance of bands at both the control (C) and test (T) lines was interpreted as a positive result; a band solely in the control line was scored as negative; and the absence of a control line, regardless of test-line visibility, invalidated the test.

### Optimization of reaction conditions for basic-RPA and RPA-LFD

For the systematic optimization of reaction conditions in the basic-RPA and RPA-LFD platforms, a series of experiments were conducted using *L. garvieae* genomic DNA as template across a temperature gradient spanning 20, 25, 30, 35, 40, 45, and 50 °C. Parallel time-course analyses were performed to determine the optimal incubation duration, with reactions carried out for 1, 5, 10, 15, 20, 25, 30, and 35 min, respectively.

### Specificity testing for basic-RPA and RPA-LFD

The specificity of both basic-RPA and RPA-LFD assays was evaluated using genomic DNA from *L. garvieae* and four related non-target pathogens: *S. agalactiae*, *A. hydrophila*, *E. piscicida*, and *S. iniae*. Each DNA sample was tested at a concentration of 1 ng per reaction under the optimized conditions. The specificity of the primers and probe was further evaluated by in silico alignment with the *L. petauri* target region (GenBank: CP141689.1), and additional specificity testing was performed using genomic DNA of *L. lactis* and *L. petauri*.

### Sensitivity analysis of basic-RPA and RPA-LFD

To assess the detection sensitivity of the basic-RPA and RPA-LFD assays, genomic DNA from *L. garvieae* and the recombinant plasmid pMD19-*adhE-ywdF* were serially diluted ten-fold. This procedure generated a genomic DNA concentration gradient from 10 ng/µL down to 1 fg/µL, and a corresponding plasmid copy-number range from 1.0 × 10^7^ to 1.0 × 10^0^ copies/µL. Aliquots (5 µL) from each dilution series served as templates for subsequent amplification in both basic-RPA and RPA-LFD detection systems.

### Preliminary applicability assessment using experimentally challenged tilapia

To evaluate the diagnostic capability of the established detection methods for *L. garvieae* infection, twenty healthy tilapia (mean weight 50 ± 10 g) were used in this experiment. Ten fish were randomly selected and intraperitoneally inoculated with 100 µL of *L. garvieae* suspension (1 × 10^6^ CFU/mL), while the remaining ten fish received an equivalent volume of phosphate‑buffered saline (PBS) as a control. Fish were monitored daily for clinical signs and mortality. At 48 h post-infection, all fish were euthanized using 500 mg/L MS-222 (MCE), regardless of clinical presentation, and their spleens were aseptically collected for total genomic DNA (including host and bacterial DNA) isolation using the HiPure Universal DNA Kit (Magen). All procedures involving animals received approval from the Laboratory Animal Centre of Huazhong Agricultural University, China (approval number HZAUFI-2024-0025). The extracted total DNA was then used as template for both basic-RPA and RPA-LFD assays to determine the infection status of challenged tilapia. Nuclease-free water was included as a negative control in each run.

## Results

### Screening and selection of RPA primers and probe

**Fig. 1:**
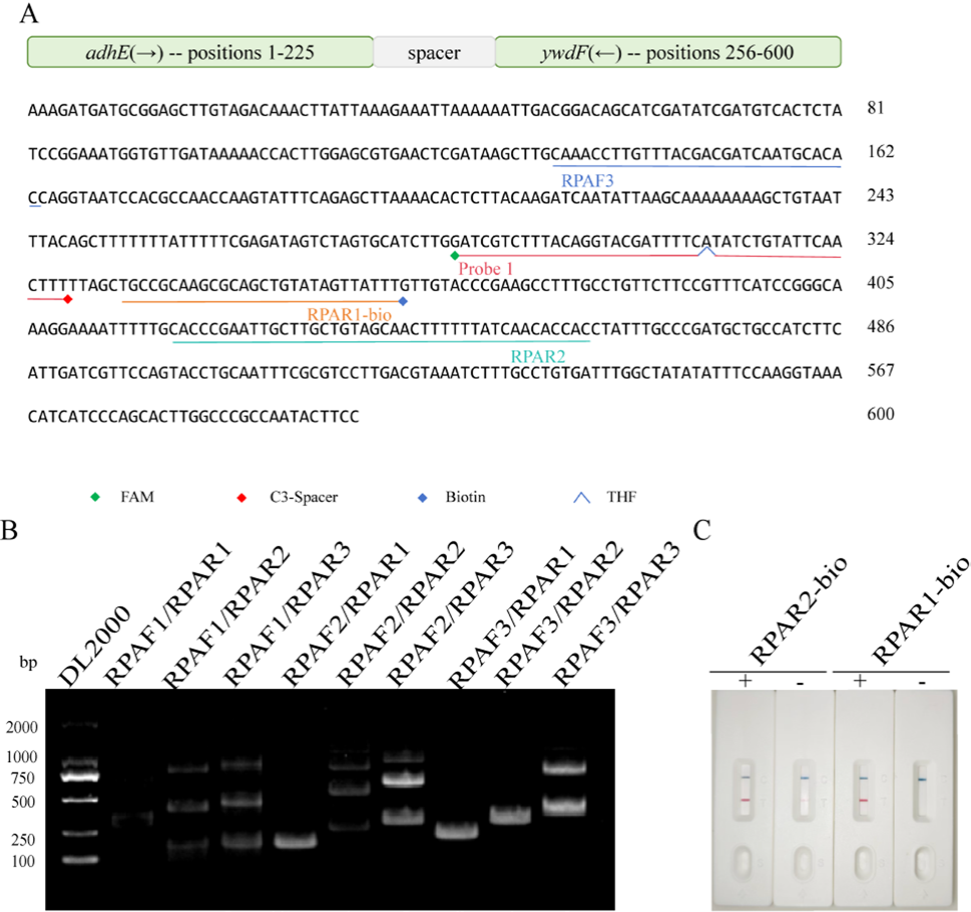
Validation of RPA primer pairs and a specific probe combination for *L. garvieae*. **A** Schematic representation of the *adhE*-*ywdF* target region. Primers and probes for RPA and RPA-LFD are underlined. **B** Amplification performance of nine candidate RPA primer pairs assessed by agarose gel electrophoresis. **C** Specificity validation of primer-nfo probe combinations. The two selected primer pairs (RPAF3/RPAR1-bio and RPAF3/RPAR2-bio) were tested with the designed nfo probe in RPA-LFD assays

Nine RPA primer pairs targeting the region spanning the 3′ end of *adhE* to the 5′ end of *ywdF* (designated *adhE-ywdF*) in *L. garvieae* were designed (Table 1). When validated with *L. garvieae* genomic DNA as template, the primer pairs RPAF3/RPAR1 and RPAF3/RPAR2 displayed enhanced amplification efficiency and were consequently chosen for further experimental work (Fig. 1 B). An nfo probe was then developed, targeting the inter-primer region flanked by the RPAF3 and RPAR1 binding sites. The specificity of this probe, combined with the two selected primer pairs, was evaluated using the RPA-LFD assay. The assay demonstrated that only the combination of RPAF3/RPAR1 with the probe yielded specific amplification without cross-reactivity or false-positive signals (Fig. 1 C). Consequently, the primer-probe set RPAF3/RPAR1 was selected for all subsequent RPA-LFD experiments.

**Table 1:** Primers and probes used for PCR, RPA and RPA-LFD

Primer name	Sequence (5′–3′)	Primer direction	Method
PCR1-F	GATGATGCGGAGCTTGTAG	Forward	
PCR1-R	CTGGGATGATGTTTACCTTGG	Reverse	
PCR2-F	ACCTTGTTTACGACGATCAATGC	Forward	PCR
PCR2-R	AAGTTGCTACAGCAAGCAATTCG	Reverse	
RPAF1	GGACAGCATCGATATCGATGTCACTCTATC	Forward	
RPAF2	CAGAGCTTAAAACACTCTTACAAGATCAAT	Forward	
RPAF3	CAAACCTTGTTTACGACGATCAATGCACAC	Forward	
RPAR1	AAATAACTATACAGCTGCGCTTGCGGCAGC	Reverse	RPA
RPAR2	GATAAAAAAGTTGCTACAGCAAGCAATTCGGGTG	Reverse	
RPAR3	TGGAACGATCAATGAAGATGGCAGCATCGG	Reverse	
Probe1	FAM/ATCTTGGATCGTCTTTACAGGTACGATTTTC(THF)TATCTGTATTCAACTTT/C3-Spacer	Probe	
RPAR1-bio	/Biotin/AAATAACTATACAGCTGCGCTTGCGGCAGC	Reverse	RPA-LFD
RPAR2-bio	/Biotin/GATAAAAAAGTTGCTACAGCAAGCAATTCGGGTG	Reverse	

### Optimization of reaction conditions for basic-RPA and RPA-LFD

**Fig. 2:**
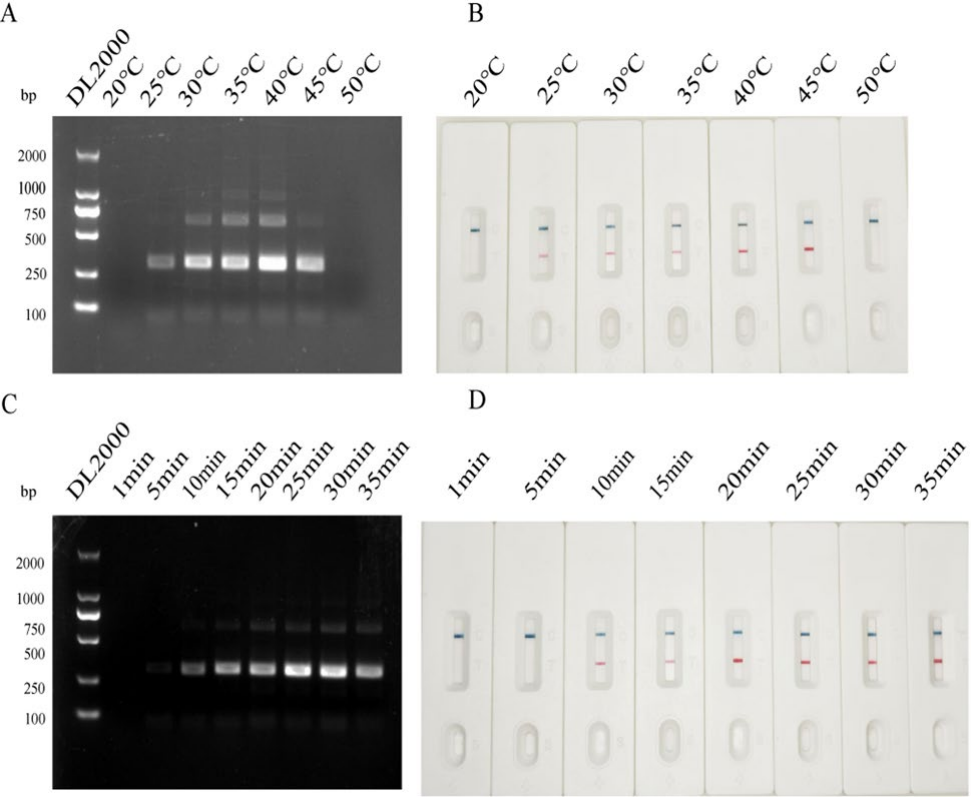
Optimization of RPA reaction conditions using the RPAF3/RPAR2 and RPAF3/RPAR1-bio/probe combinations. **A** Agarose gel electrophoresis analysis of RPA products amplified at different temperatures. **B** LFD detection of RPA products generated at various temperatures. **C** Agarose gel electrophoresis analysis of RPA products obtained at different reaction times. **D** LFD detection of RPA products after different reaction durations. *L. garvieae* genomic DNA served as the template in all assays

Aiming to define the most favorable reaction parameters, amplification efficiency was initially assessed across a temperature gradient spanning 20 °C to 50 °C. The data indicated that robust amplification for both basic-RPA and RPA-LFD assays was achieved across a broad thermal range of 25–45 °C, with peak efficiency consistently recorded at 40 °C (Fig. 2 A and B). Consequently, 40 °C was designated as the optimal incubation temperature for all subsequent assays.

Following this, the ideal incubation time was determined through testing amplification over durations ranging from 1 to 35 min. Detectable amplification for both basic-RPA and RPA-LFD was achieved within 10–35 min (Fig. 2 C and D). Based on these results, a 15-minute incubation period was adopted as the standard duration for all subsequent experimental runs.

### Specificity of the basic-RPA and RPA-LFD assays

**Fig. 3:**
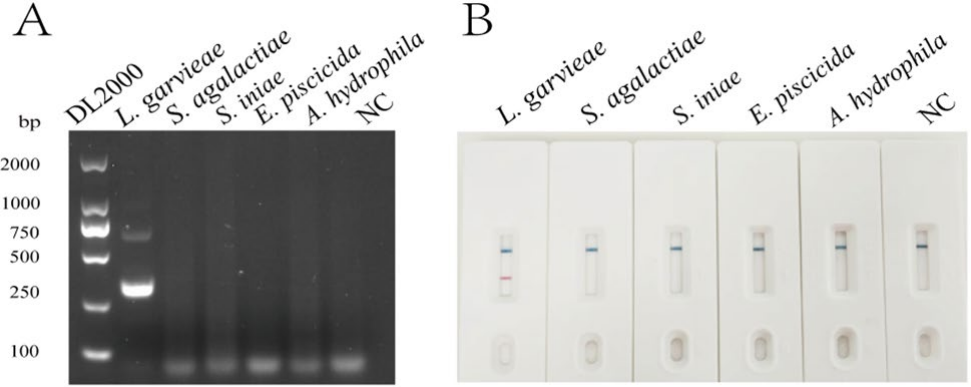
Specificity assessment of basic-RPA and RPA-LFD assays against prevalent fish-associated bacteria. Genomic DNA from *L. garvieae*, *S. agalactiae*, *S. iniae*, *E. piscicida*, and *A. hydrophila* was used as template, alongside a nuclease-free water negative control. **A** Agarose gel electrophoretic analysis of RPA amplicons. **B** Verification of RPA amplification products by LFD. NC, negative control

The specificity of both the basic-RPA and RPA-LFD assays was evaluated using genomic DNA from *L. garvieae* and several common fish pathogenic bacteria, including *S. agalactiae*, *S. iniae*, *E. piscicida*, and *A. hydrophila* as templates under the optimized reaction conditions. Specific amplification was observed exclusively in reactions containing *L. garvieae* genomic DNA, whereas all non-target templates yielded negative results (Fig. 3 A and B). The *adhE*-*ywdF* target region was highly conserved among the tested *L. garvieae* strains, as shown by multiple sequence alignment (Supplementary Fig. S2). Additional testing showed that the RPA-LFD assay also gave negative results for *L. lactis* (Supplementary Fig. S3B), indicating genus-level specificity.

However, when tested against *L. petauri*, the assay produced clear positive signals (Supplementary Fig. S3B). In silico alignment of the primer and probe binding sites with the *L. petauri* genome revealed high sequence similarity (Supplementary Fig. S3A). Therefore, the RPA-LFD assay does not discriminate between *L. garvieae* and *L. petauri*. Given that both species cause clinically indistinguishable lactococcosis and are important pathogens in tilapia, the method is suitable as a rapid screening tool for lactococcosis outbreaks rather than a species-specific diagnostic.

### Sensitivity of the basic-RPA and RPA-LFD assays

**Fig. 4:**
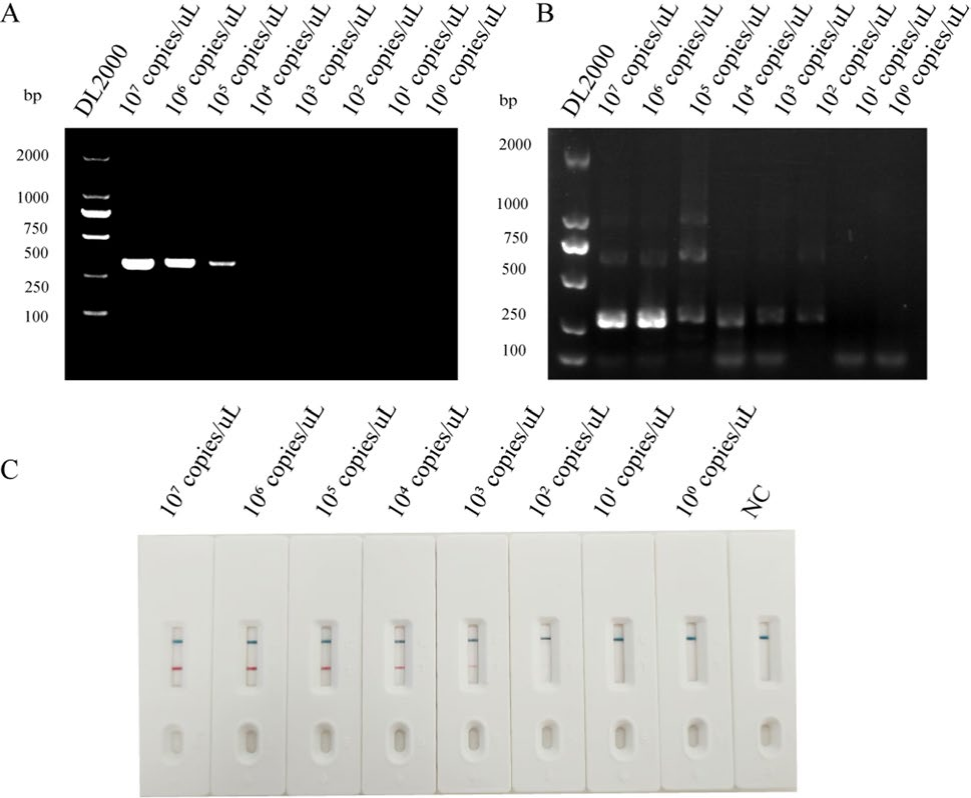
Detection sensitivity evaluation of RPA assays with plasmid pMD19-*adhE*‑*ywdF* as template. PCR, RPA and RPA-LFD assays were performed with serially diluted pMD19-*adhE-ywdF* plasmid covering a range of copy numbers. Nuclease-free water was included as the negative control. **A** Agarose gel electrophoresis analysis of PCR products. **B** Agarose gel electrophoresis analysis of RPA products. **C** LFD detection of RPA products

**Fig. 5:**
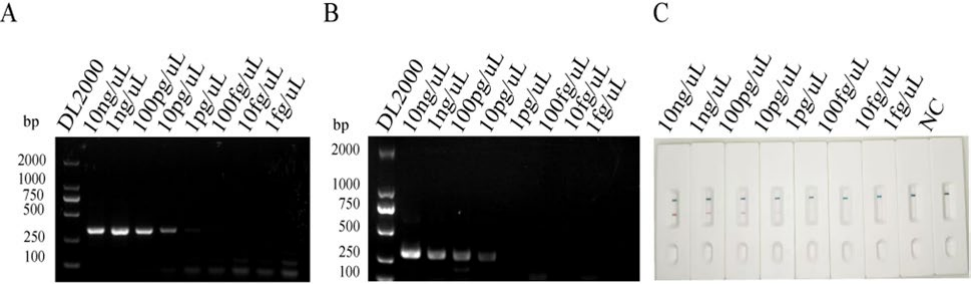
Analytical sensitivity of RPA methods assessed with *L. garvieae* genomic DNA as template. PCR, RPA and RPA-LFD assays were performed using serially diluted *L. garvieae* genomic DNA. Nuclease-free water served as the negative control. **A** Agarose gel electrophoresis of PCR products. **B** Agarose gel electrophoresis of RPA products. **C** LFD detection of RPA products

Under the optimized conditions, the sensitivity of the assays was evaluated using serially diluted pMD19-*adhE-ywdF* plasmid as template. Both RPA and RPA-LFD successfully detected the target across a plasmid concentration range of 10^3^ to 10^7^ copies/µL, exhibiting a lower detection threshold of 10^3^ copies/µL. In contrast, conventional PCR showed a higher detection limit of 10^5^ copies/µL under the same conditions (Fig. 4 ). A parallel sensitivity assessment was performed using serially diluted *L. garvieae* genomic DNA (ranging from 10 ng/µL to 1 fg/µL). All three methods, including PCR, RPA, and RPA-LFD, consistently exhibited a detection limit of 10 pg/µL (Fig. 5 ).

### Detection performance on samples from experimentally challenged tilapia

**Fig. 6:**
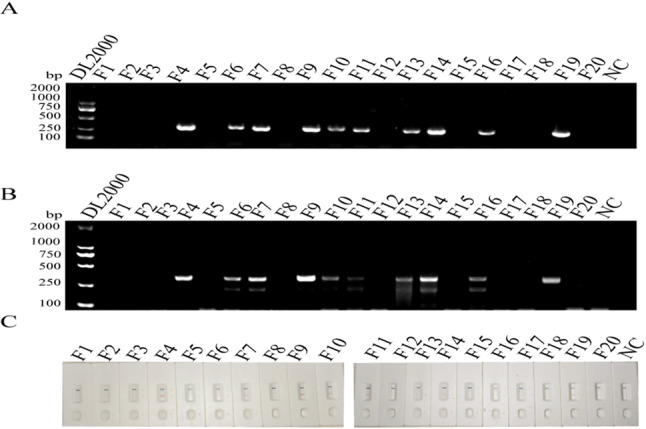
Detection performance of the established RPA assays on samples from experimentally challenged tilapia. Spleen-derived DNA extracted from 20 experimental samples (F1-F20) was used as the template for PCR, RPA and RPA-LFD assays, using nuclease-free water included as the negative control. **A** Agarose gel electrophoresis analysis of PCR products. **B** Agarose gel electrophoresis analysis of RPA products. **C** LFD detection of RPA products

To evaluate the detection performance of the established methods under controlled conditions, we analyzed spleen-derived DNA from 20 tilapia specimens. To simulate a blind detection scenario, the infected and control fish were mixed before sampling. Some fish showed various degrees of lactococcosis signs (less than 10 fish), including corneal opacity, abdominal distension, and skin hemorrhages, but it was impossible to distinguish the infected group from the controls based solely on clinical signs. Detection by both basic-RPA and RPA-LFD correctly identified all 10 infected samples as positive and all 10 control samples as negative. The results showed complete concordance with conventional PCR (Fig. 6 ). Bacterial re-isolation from spleen and kidney confirmed systemic infection in all 10 infected fish. These results provide initial evidence for the potential utility of the methods as a rapid screening tool for lactococcosis under controlled conditions. Larger-scale studies with naturally infected field samples are needed to further validate the diagnostic performance.

## Discussion

Lactococcosis is a serious bacterial disease in fish, caused by several *Lactococcus* species, and has led to significant economic losses in the aquaculture industry [1]. The clinical signs of lactococcosis, which include erratic swimming, exophthalmia, corneal opacity, ascites, and hemorrhages in visceral organs and skin, often overlap with those of streptococcosis. This overlap makes differential diagnosis based solely on clinical observation challenging [5]. To address this diagnostic challenge, we developed both basic-RPA and RPA-LFD assays for the rapid and reliable detection of piscine lactococcosis. RPA-based methods are known for their operational simplicity, high sensitivity, and short turnaround time [18, 20, 25], and have been widely applied in screening diverse bacterial, viral, and parasitic pathogens [23, 25–27]. To the best of our knowledge, this work represents the initial documentation of RPA-based methodology applied to the detection of lactococcosis.

Currently, the predominant methods for detecting *L. garvieae*, such as PCR, qPCR, and LAMP, rely on thermal cycling or sustained heating along with specialized instrumentation [11–13], making them poorly adapted for rapid, on-site use in resource-limited settings like aquaculture farms. In addition, some widely used PCR primer sets targeting regions such as the internal transcribed spacer (ITS) of 16–23 S rRNA showed false positive results in non-*L. garvieae* species [14, 15] (Fig. S1). To address these limitations, we employed comparative genomic analysis to identify target sequences within *L. garvieae* and we developed detection assays based on basic-RPA and RPA-LFD. The resulting RPA-LFD platform addresses these limitations by integrating isothermal amplification with instrument-free visual detection. Its main operational advantages include: (1) low-temperature requirement (effective from 25 to 45 °C), enabling amplification with ambient heat or simple portable devices. (2) Quickly visualize the readings through the lateral flow dipstick within minutes [18, 22, 28]. The compact and user-friendly design of this equipment significantly reduces technical barriers and enhances on-site applicability. Notably, in the present experimental challenge, some infected fish exhibited no obvious clinical signs yet tested positive by both RPA and RPA-LFD, highlighting the value of a molecular screening tool that does not rely on visible symptoms for early detection. However, this study was designed as a proof-of-concept, and several limitations should be noted. The validation used only 20 experimentally infected fish (10 infected, 10 controls), which is insufficient for full diagnostic performance evaluation under field conditions. Future work should include larger-scale testing with naturally infected fish from different farms and disease stages to confirm the method’s robustness and generalizability.

In addition, we further evaluated the specificity towards other *Lactococcus* species. The RPA-LFD assay gave a negative result for *L. lactis*, indicating genus-level specificity. However, in silico alignment of the primer and probe binding sites with the *L. petauri* genome revealed high sequence similarity. Consistent with this, the RPA-LFD assay produced positive signals for *L. petauri* (Supplementary Fig. S3). This is not unexpected, because *L. petauri* was only formally recognized as a new species in 2017 and is phylogenetically very close to *L. garvieae*; indeed, routine molecular methods such as 16S rRNA sequencing cannot reliably distinguish the two species [29]. Therefore, the method does not discriminate between *L. garvieae* and *L. petauri*. Importantly, *L. petauri* has recently been confirmed as a major causative agent of lactococcosis in Nile tilapia, causing similar clinical signs and economic losses. Given that both species cause clinically indistinguishable lactococcosis, the method remains useful as a rapid screening tool for lactococcosis outbreaks but is not suitable for species-level diagnosis [30]. For species identification, complementary methods are required.

For on-site epidemic investigation, this platform may serve as a useful frontline screening tool to rapidly detect lactococcosis during acute mortality events, enabling timely intervention [31, 32]. It is equally applicable to asymptomatic surveillance, such as the routine monitoring of broodstock, fry, or pond water [19, 31]. Given the acute nature and high mortality rate of lactococcosis [1, 2], such early detection is particularly valuable for issuing preemptive warnings and strengthening biosecurity management. Furthermore, the method could be adapted for port-of-entry and border inspection, offering a rapid means to help prevent the transboundary spread of the pathogen.

Despite these advantages, several aspects warrant further development to fully realize the platform’s potential. First, the current assay depends on extracted DNA; integrating it with simple, rapid nucleic acid release methods would enable a fully integrated field-deployable workflow, significantly improving field utility [33]. Second, given the frequency of co‑infections in aquaculture, future work could focus on developing a multiplex RPA‑LFD strip capable of simultaneously differentiating major pathogens like *S. agalactiae* and *S. iniae*, thereby enhancing diagnostic throughput and clinical relevance [34, 35]. Finally, coupling the RPA reaction with a portable fluorescence or colorimetric reader could convert the assay from a qualitative to a semi‑quantitative or quantitative platform, offering valuable data on pathogen load for monitoring disease progression or assessing treatment efficacy [36–38].

## Conclusions

In summary, this study has established and validated rapid visual assays for lactococcosis*-*associated *Lactococcus* species based on basic‑RPA and RPA‑LFD. The developed platform overcomes major limitations of conventional techniques by enabling isothermal amplification (25–45 °C) and instrument‑free visual readout. The assays showed a detection limit of 10^3^ copies/µL for plasmid DNA and 10 pg/µL for genomic DNA, and were specific against the tested non-target pathogens. Cross-reactivity with *L. petauri* was noted, but this does not diminish the assay’s utility for lactococcosis outbreak screening. This preliminary proof-of-concept evaluation showed excellent agreement with standard methods, suggesting diagnostic reliability. These attributes suggest that the platform could be useful for on-site screening of lactococcosis outbreaks. Nevertheless, the current evaluation was based on a small number of experimentally infected samples, and large-scale validation using field-collected samples from natural outbreaks is still needed to confirm the practical diagnostic performance in aquaculture settings. Future integration with simplified sample preparation (currently still dependent on kit-based DNA extraction) and multiplex detection may further improve its utility for aquatic disease surveillance and biosecurity.

## Supplementary Information

Supplementary Material 1. Supplementary Fig. S1. Specificity assessment of two previously published PCR primer sets for L. garvieae detection. Agarose gel electrophoresis analysis of PCR products amplified from genomic DNA of various bacterial pathogens. (A) PCR using the 16S-23S rRNA ITS-targeting primer set. (B) PCR using the 16S rRNA-targeting primer set. Supplementary Fig. S2. Multiple sequence alignment of the adhE-ywdF target region among fish-derived L. garvieae strains. The aligned sequences include four L. garvieae strains isolated from fish: AP027239.1, AP009333.1, AP009332.1 and AP043994.1. The target region showed 100% nucleotide identity across all tested strains, confirming that the selected target is highly conserved within L. garvieae. Supplementary Fig. S3. Sequence alignment of the target region and RPA-LFD detection of Lactococcus species. (A) Partial nucleotide sequence alignment of the adhE-ywdF target region from L. garvieae and L. petauri. The binding sites of the forward primer RPAF3, the reverse primer RPAR1-bio, and the nfo probe are indicated. (B) RPA-LFD assay results using genomic DNA from L. garvieae, L. petauri, L. lactis, and a no‑template control (NC). Positive signals (test line) were obtained for both L. garvieae and L. petauri, indicating cross‑reactivity. No amplification was observed for L. lactis or the NC.

## Data Availability

The genome sequences of Lactococcus garvieae analyzed in this study are publicly available in the NCBI GenBank repository under accession numbers AP027239.1, AP009332.1, AP009333.1, and AP043994.1 (https://www.ncbi.nlm.nih.gov/nuccore/AP027239.1; https://www.ncbi.nlm.nih.gov/nuccore/AP009332.1; https://www.ncbi.nlm.nih.gov/nuccore/AP009333.1; https://www.ncbi.nlm.nih.gov/nuccore/AP043994.1). The genome sequence of Lactococcus petauri is available under accession number CP141689.1 (https://www.ncbi.nlm.nih.gov/nuccore/CP141689.1). The genome sequence of Streptococcus agalactiae is available under accession number CP011325.1 (https://www.ncbi.nlm.nih.gov/nuccore/CP011325.1). All other data generated or analyzed during this study are included in this published article.
