# Supplementary figures and images for: Establishment of methods for visual and rapid detection of piscine lactococcosis based on isothermal recombinase polymerase amplification
Source: BMC Vet Res. 2026 May 28;22:448. doi: 10.1186/s12917-026-05587-5 (PMC13418454; doi:10.1186/s12917-026-05587-5)

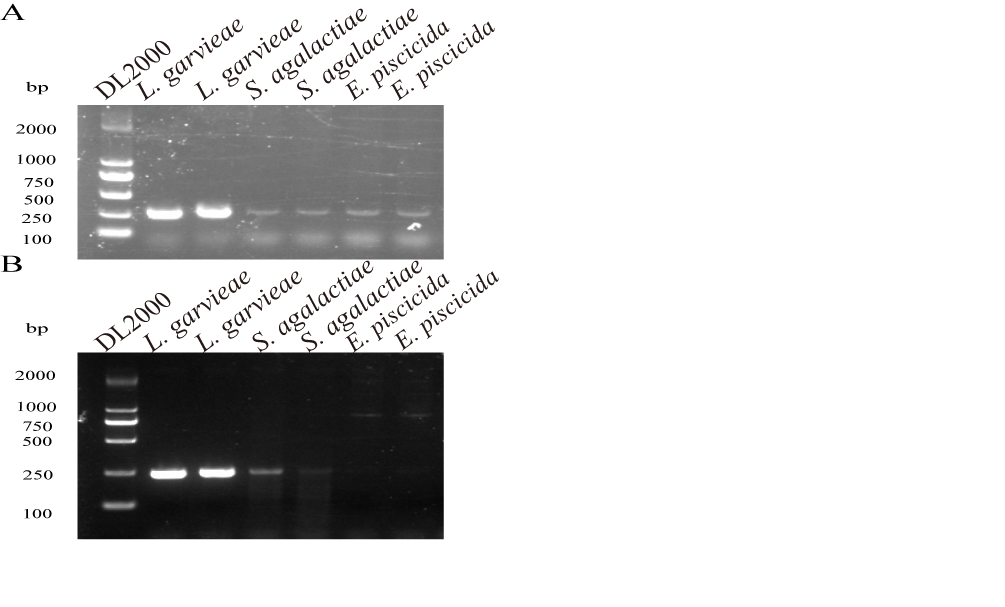

Supplement: Supplementary file 1 — Supplementary Material 1. Supplementary Fig. S1. Specificity assessment of two previously published PCR primer sets for L. garvieae detection. Agarose gel electrophoresis analysis of PCR products amplified from genomic DNA of various bacterial pathogens. (A) PCR using the 16S-23S rRNA ITS-targeting primer set. (B) PCR using the 16S rRNA-targeting primer set. Supplementary Fig. S2. Multiple sequence alignment of the adhE-ywdF target region among fish-derived L. garvieae strains. The aligned sequences include four L. garvieae strains isolated from fish: AP027239.1, AP009333.1, AP009332.1 and AP043994.1. The target region showed 100% nucleotide identity across all tested strains, confirming that the selected target is highly conserved within L. garvieae. Supplementary Fig. S3. Sequence alignment of the target region and RPA-LFD detection of Lactococcus species. (A) Partial nucleotide sequence alignment of the adhE-ywdF target region from L. garvieae and L. petauri. The binding sites of the forward primer RPAF3, the reverse primer RPAR1-bio, and the nfo probe are indicated. (B) RPA-LFD assay results using genomic DNA from L. garvieae, L. petauri, L. lactis, and a no‑template control (NC). Positive signals (test line) were obtained for both L. garvieae and L. petauri, indicating cross‑reactivity. No amplification was observed for L. lactis or the NC. [file 12917_2026_5587_MOESM1_ESM.zip › 12917_2026_5587_MOESM1_ESM.tif]

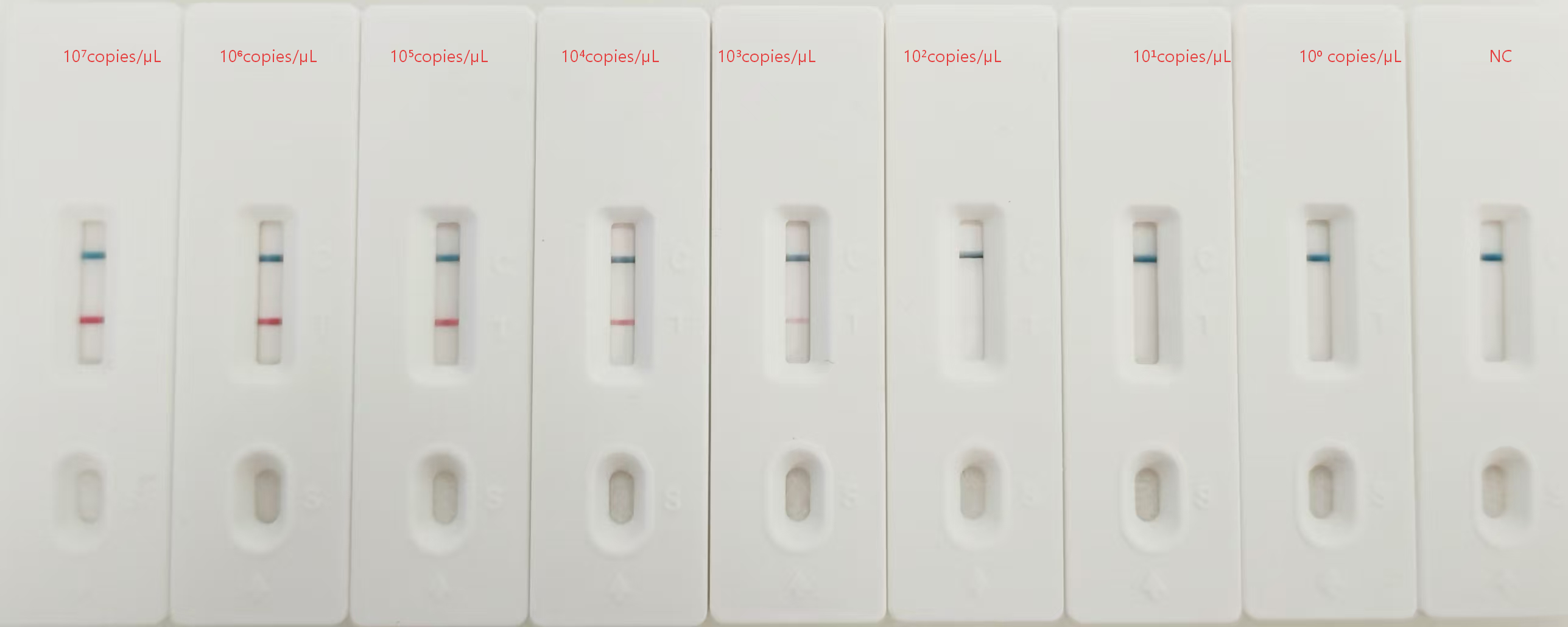

Supplement: Supplementary file 1 — Supplementary Material 1. Supplementary Fig. S1. Specificity assessment of two previously published PCR primer sets for L. garvieae detection. Agarose gel electrophoresis analysis of PCR products amplified from genomic DNA of various bacterial pathogens. (A) PCR using the 16S-23S rRNA ITS-targeting primer set. (B) PCR using the 16S rRNA-targeting primer set. Supplementary Fig. S2. Multiple sequence alignment of the adhE-ywdF target region among fish-derived L. garvieae strains. The aligned sequences include four L. garvieae strains isolated from fish: AP027239.1, AP009333.1, AP009332.1 and AP043994.1. The target region showed 100% nucleotide identity across all tested strains, confirming that the selected target is highly conserved within L. garvieae. Supplementary Fig. S3. Sequence alignment of the target region and RPA-LFD detection of Lactococcus species. (A) Partial nucleotide sequence alignment of the adhE-ywdF target region from L. garvieae and L. petauri. The binding sites of the forward primer RPAF3, the reverse primer RPAR1-bio, and the nfo probe are indicated. (B) RPA-LFD assay results using genomic DNA from L. garvieae, L. petauri, L. lactis, and a no‑template control (NC). Positive signals (test line) were obtained for both L. garvieae and L. petauri, indicating cross‑reactivity. No amplification was observed for L. lactis or the NC. [file 12917_2026_5587_MOESM1_ESM.zip › 12917_2026_5587_MOESM10_ESM.png]

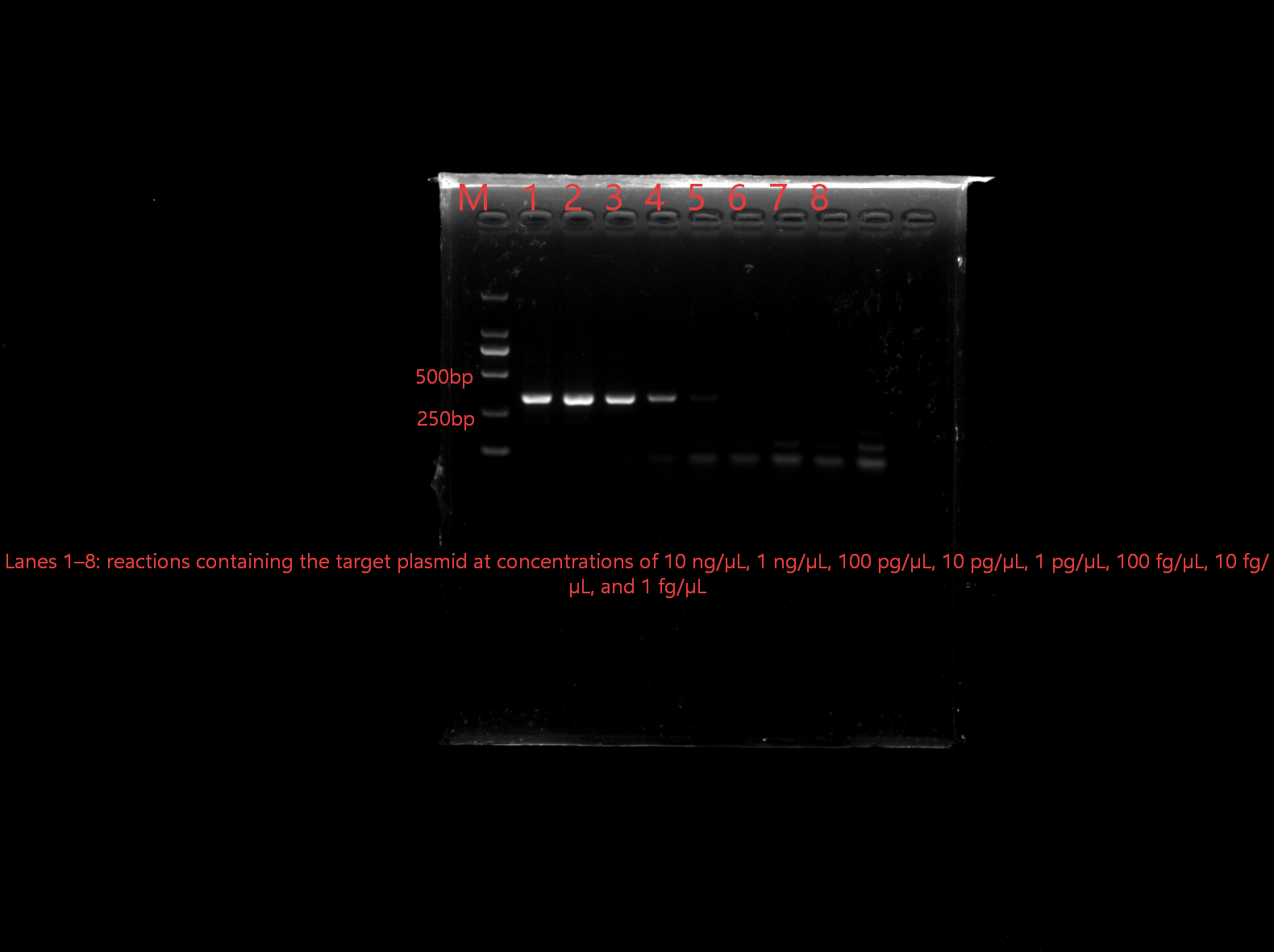

Supplement: Supplementary file 1 — Supplementary Material 1. Supplementary Fig. S1. Specificity assessment of two previously published PCR primer sets for L. garvieae detection. Agarose gel electrophoresis analysis of PCR products amplified from genomic DNA of various bacterial pathogens. (A) PCR using the 16S-23S rRNA ITS-targeting primer set. (B) PCR using the 16S rRNA-targeting primer set. Supplementary Fig. S2. Multiple sequence alignment of the adhE-ywdF target region among fish-derived L. garvieae strains. The aligned sequences include four L. garvieae strains isolated from fish: AP027239.1, AP009333.1, AP009332.1 and AP043994.1. The target region showed 100% nucleotide identity across all tested strains, confirming that the selected target is highly conserved within L. garvieae. Supplementary Fig. S3. Sequence alignment of the target region and RPA-LFD detection of Lactococcus species. (A) Partial nucleotide sequence alignment of the adhE-ywdF target region from L. garvieae and L. petauri. The binding sites of the forward primer RPAF3, the reverse primer RPAR1-bio, and the nfo probe are indicated. (B) RPA-LFD assay results using genomic DNA from L. garvieae, L. petauri, L. lactis, and a no‑template control (NC). Positive signals (test line) were obtained for both L. garvieae and L. petauri, indicating cross‑reactivity. No amplification was observed for L. lactis or the NC. [file 12917_2026_5587_MOESM1_ESM.zip › 12917_2026_5587_MOESM11_ESM.tif]

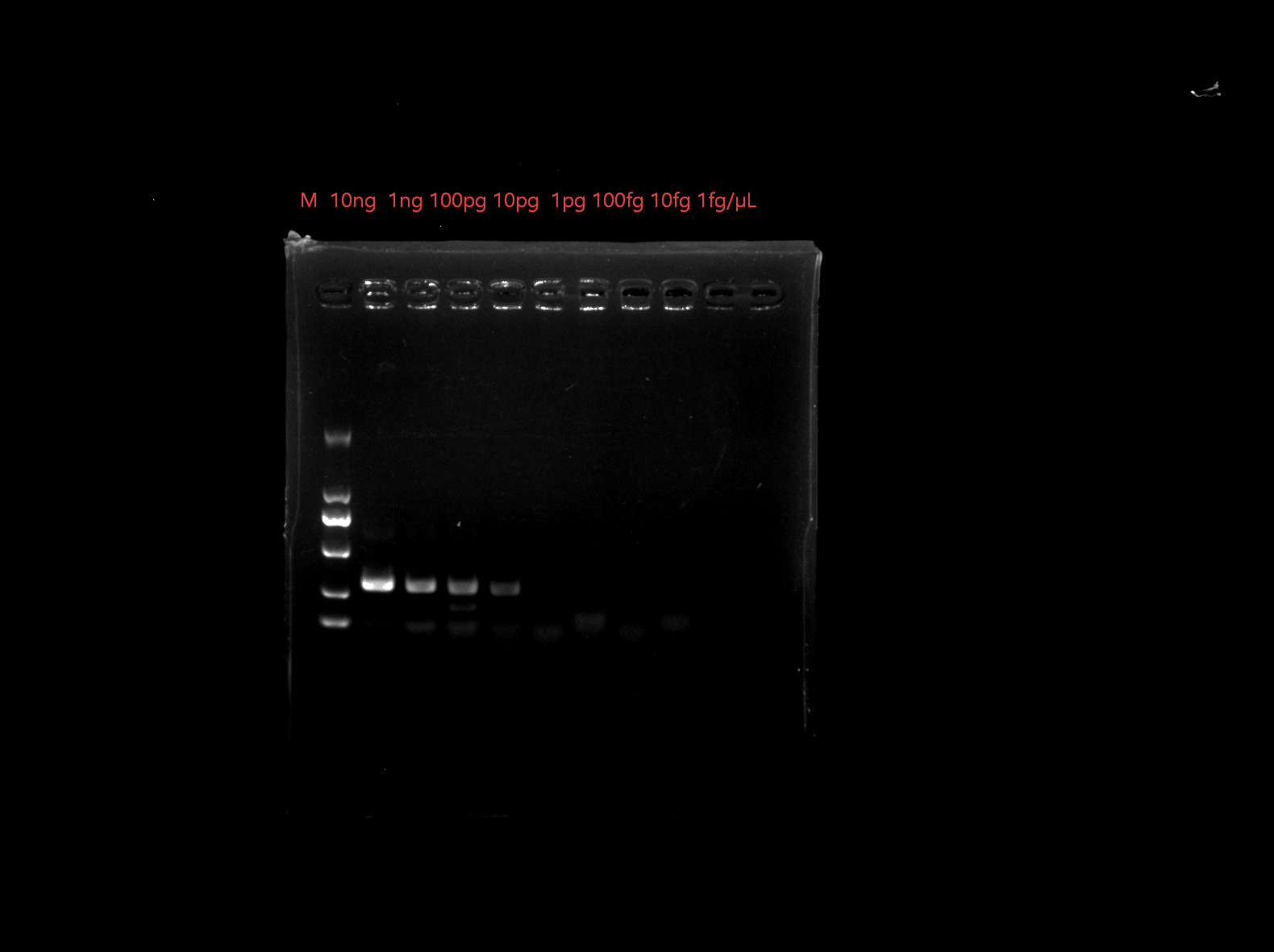

Supplement: Supplementary file 1 — Supplementary Material 1. Supplementary Fig. S1. Specificity assessment of two previously published PCR primer sets for L. garvieae detection. Agarose gel electrophoresis analysis of PCR products amplified from genomic DNA of various bacterial pathogens. (A) PCR using the 16S-23S rRNA ITS-targeting primer set. (B) PCR using the 16S rRNA-targeting primer set. Supplementary Fig. S2. Multiple sequence alignment of the adhE-ywdF target region among fish-derived L. garvieae strains. The aligned sequences include four L. garvieae strains isolated from fish: AP027239.1, AP009333.1, AP009332.1 and AP043994.1. The target region showed 100% nucleotide identity across all tested strains, confirming that the selected target is highly conserved within L. garvieae. Supplementary Fig. S3. Sequence alignment of the target region and RPA-LFD detection of Lactococcus species. (A) Partial nucleotide sequence alignment of the adhE-ywdF target region from L. garvieae and L. petauri. The binding sites of the forward primer RPAF3, the reverse primer RPAR1-bio, and the nfo probe are indicated. (B) RPA-LFD assay results using genomic DNA from L. garvieae, L. petauri, L. lactis, and a no‑template control (NC). Positive signals (test line) were obtained for both L. garvieae and L. petauri, indicating cross‑reactivity. No amplification was observed for L. lactis or the NC. [file 12917_2026_5587_MOESM1_ESM.zip › 12917_2026_5587_MOESM12_ESM.tif]

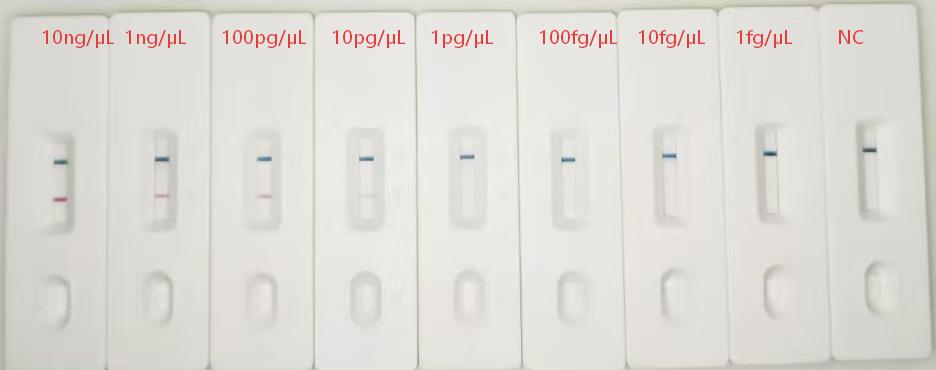

Supplement: Supplementary file 1 — Supplementary Material 1. Supplementary Fig. S1. Specificity assessment of two previously published PCR primer sets for L. garvieae detection. Agarose gel electrophoresis analysis of PCR products amplified from genomic DNA of various bacterial pathogens. (A) PCR using the 16S-23S rRNA ITS-targeting primer set. (B) PCR using the 16S rRNA-targeting primer set. Supplementary Fig. S2. Multiple sequence alignment of the adhE-ywdF target region among fish-derived L. garvieae strains. The aligned sequences include four L. garvieae strains isolated from fish: AP027239.1, AP009333.1, AP009332.1 and AP043994.1. The target region showed 100% nucleotide identity across all tested strains, confirming that the selected target is highly conserved within L. garvieae. Supplementary Fig. S3. Sequence alignment of the target region and RPA-LFD detection of Lactococcus species. (A) Partial nucleotide sequence alignment of the adhE-ywdF target region from L. garvieae and L. petauri. The binding sites of the forward primer RPAF3, the reverse primer RPAR1-bio, and the nfo probe are indicated. (B) RPA-LFD assay results using genomic DNA from L. garvieae, L. petauri, L. lactis, and a no‑template control (NC). Positive signals (test line) were obtained for both L. garvieae and L. petauri, indicating cross‑reactivity. No amplification was observed for L. lactis or the NC. [file 12917_2026_5587_MOESM1_ESM.zip › 12917_2026_5587_MOESM13_ESM.jpg]

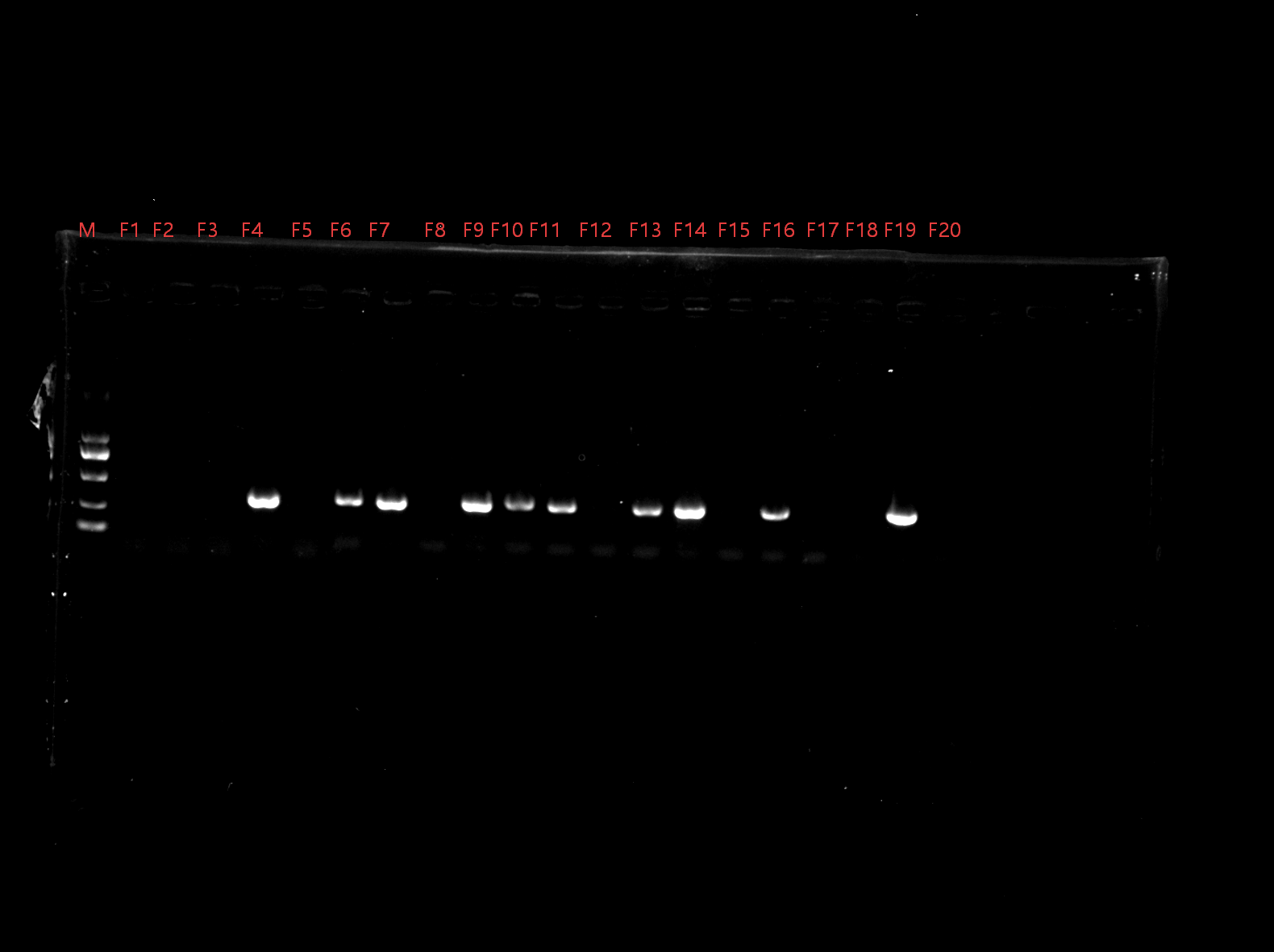

Supplement: Supplementary file 1 — Supplementary Material 1. Supplementary Fig. S1. Specificity assessment of two previously published PCR primer sets for L. garvieae detection. Agarose gel electrophoresis analysis of PCR products amplified from genomic DNA of various bacterial pathogens. (A) PCR using the 16S-23S rRNA ITS-targeting primer set. (B) PCR using the 16S rRNA-targeting primer set. Supplementary Fig. S2. Multiple sequence alignment of the adhE-ywdF target region among fish-derived L. garvieae strains. The aligned sequences include four L. garvieae strains isolated from fish: AP027239.1, AP009333.1, AP009332.1 and AP043994.1. The target region showed 100% nucleotide identity across all tested strains, confirming that the selected target is highly conserved within L. garvieae. Supplementary Fig. S3. Sequence alignment of the target region and RPA-LFD detection of Lactococcus species. (A) Partial nucleotide sequence alignment of the adhE-ywdF target region from L. garvieae and L. petauri. The binding sites of the forward primer RPAF3, the reverse primer RPAR1-bio, and the nfo probe are indicated. (B) RPA-LFD assay results using genomic DNA from L. garvieae, L. petauri, L. lactis, and a no‑template control (NC). Positive signals (test line) were obtained for both L. garvieae and L. petauri, indicating cross‑reactivity. No amplification was observed for L. lactis or the NC. [file 12917_2026_5587_MOESM1_ESM.zip › 12917_2026_5587_MOESM14_ESM.tif]

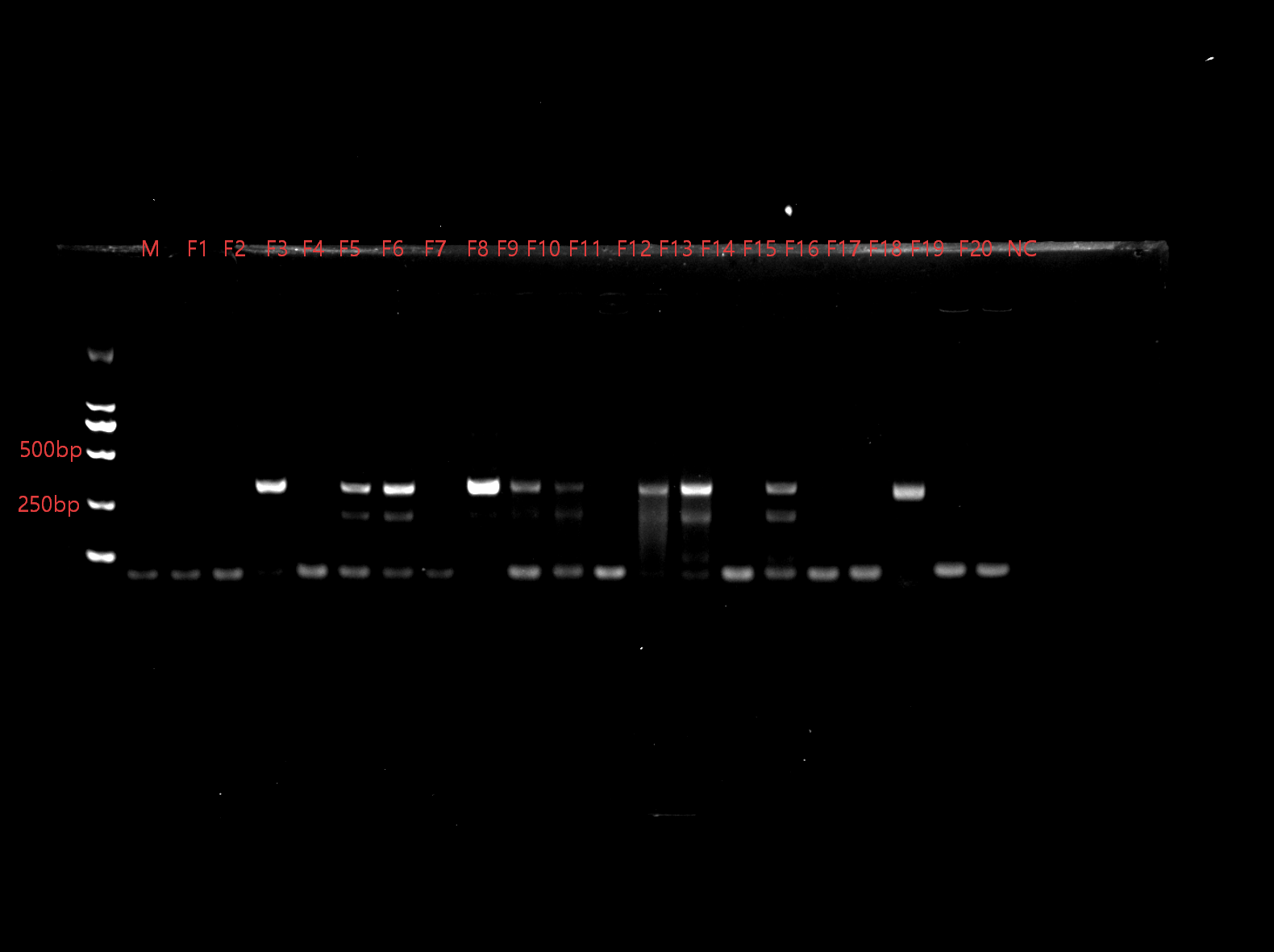

Supplement: Supplementary file 1 — Supplementary Material 1. Supplementary Fig. S1. Specificity assessment of two previously published PCR primer sets for L. garvieae detection. Agarose gel electrophoresis analysis of PCR products amplified from genomic DNA of various bacterial pathogens. (A) PCR using the 16S-23S rRNA ITS-targeting primer set. (B) PCR using the 16S rRNA-targeting primer set. Supplementary Fig. S2. Multiple sequence alignment of the adhE-ywdF target region among fish-derived L. garvieae strains. The aligned sequences include four L. garvieae strains isolated from fish: AP027239.1, AP009333.1, AP009332.1 and AP043994.1. The target region showed 100% nucleotide identity across all tested strains, confirming that the selected target is highly conserved within L. garvieae. Supplementary Fig. S3. Sequence alignment of the target region and RPA-LFD detection of Lactococcus species. (A) Partial nucleotide sequence alignment of the adhE-ywdF target region from L. garvieae and L. petauri. The binding sites of the forward primer RPAF3, the reverse primer RPAR1-bio, and the nfo probe are indicated. (B) RPA-LFD assay results using genomic DNA from L. garvieae, L. petauri, L. lactis, and a no‑template control (NC). Positive signals (test line) were obtained for both L. garvieae and L. petauri, indicating cross‑reactivity. No amplification was observed for L. lactis or the NC. [file 12917_2026_5587_MOESM1_ESM.zip › 12917_2026_5587_MOESM15_ESM.tif]

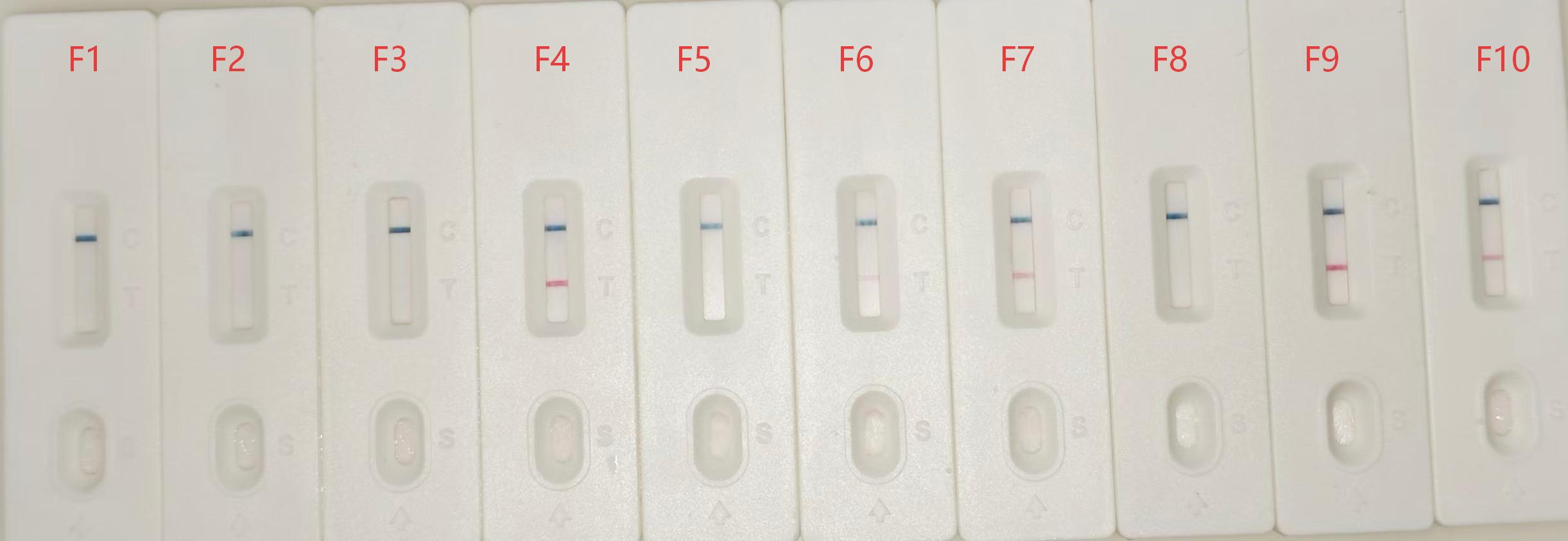

Supplement: Supplementary file 1 — Supplementary Material 1. Supplementary Fig. S1. Specificity assessment of two previously published PCR primer sets for L. garvieae detection. Agarose gel electrophoresis analysis of PCR products amplified from genomic DNA of various bacterial pathogens. (A) PCR using the 16S-23S rRNA ITS-targeting primer set. (B) PCR using the 16S rRNA-targeting primer set. Supplementary Fig. S2. Multiple sequence alignment of the adhE-ywdF target region among fish-derived L. garvieae strains. The aligned sequences include four L. garvieae strains isolated from fish: AP027239.1, AP009333.1, AP009332.1 and AP043994.1. The target region showed 100% nucleotide identity across all tested strains, confirming that the selected target is highly conserved within L. garvieae. Supplementary Fig. S3. Sequence alignment of the target region and RPA-LFD detection of Lactococcus species. (A) Partial nucleotide sequence alignment of the adhE-ywdF target region from L. garvieae and L. petauri. The binding sites of the forward primer RPAF3, the reverse primer RPAR1-bio, and the nfo probe are indicated. (B) RPA-LFD assay results using genomic DNA from L. garvieae, L. petauri, L. lactis, and a no‑template control (NC). Positive signals (test line) were obtained for both L. garvieae and L. petauri, indicating cross‑reactivity. No amplification was observed for L. lactis or the NC. [file 12917_2026_5587_MOESM1_ESM.zip › 12917_2026_5587_MOESM16_ESM.jpg]

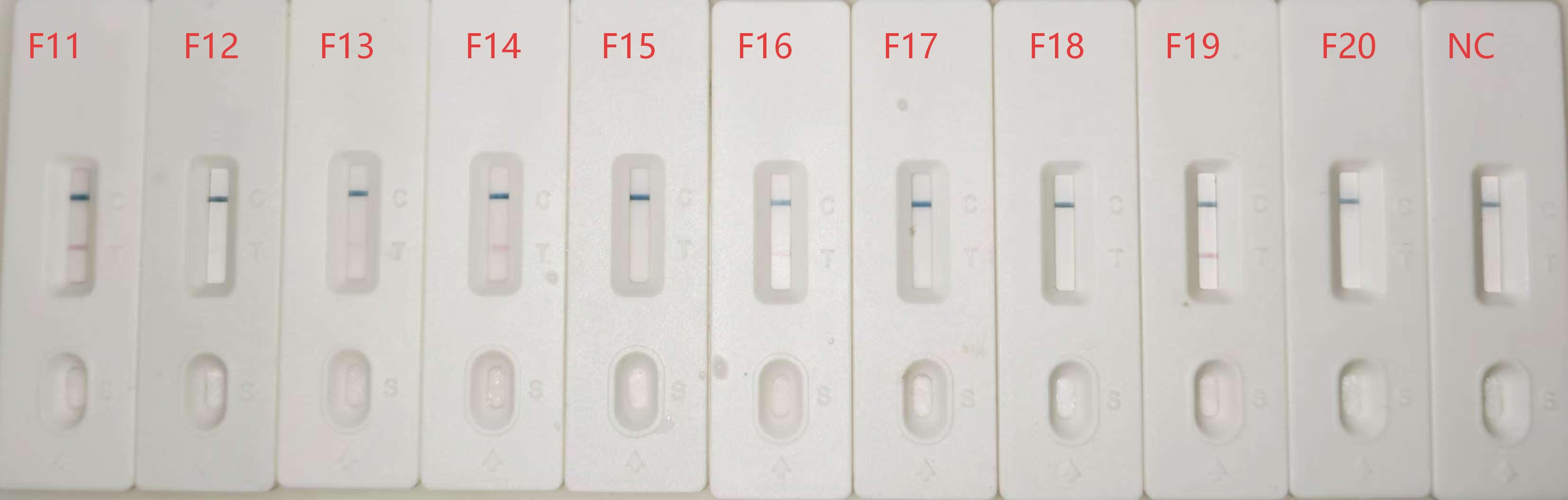

Supplement: Supplementary file 1 — Supplementary Material 1. Supplementary Fig. S1. Specificity assessment of two previously published PCR primer sets for L. garvieae detection. Agarose gel electrophoresis analysis of PCR products amplified from genomic DNA of various bacterial pathogens. (A) PCR using the 16S-23S rRNA ITS-targeting primer set. (B) PCR using the 16S rRNA-targeting primer set. Supplementary Fig. S2. Multiple sequence alignment of the adhE-ywdF target region among fish-derived L. garvieae strains. The aligned sequences include four L. garvieae strains isolated from fish: AP027239.1, AP009333.1, AP009332.1 and AP043994.1. The target region showed 100% nucleotide identity across all tested strains, confirming that the selected target is highly conserved within L. garvieae. Supplementary Fig. S3. Sequence alignment of the target region and RPA-LFD detection of Lactococcus species. (A) Partial nucleotide sequence alignment of the adhE-ywdF target region from L. garvieae and L. petauri. The binding sites of the forward primer RPAF3, the reverse primer RPAR1-bio, and the nfo probe are indicated. (B) RPA-LFD assay results using genomic DNA from L. garvieae, L. petauri, L. lactis, and a no‑template control (NC). Positive signals (test line) were obtained for both L. garvieae and L. petauri, indicating cross‑reactivity. No amplification was observed for L. lactis or the NC. [file 12917_2026_5587_MOESM1_ESM.zip › 12917_2026_5587_MOESM17_ESM.jpg]

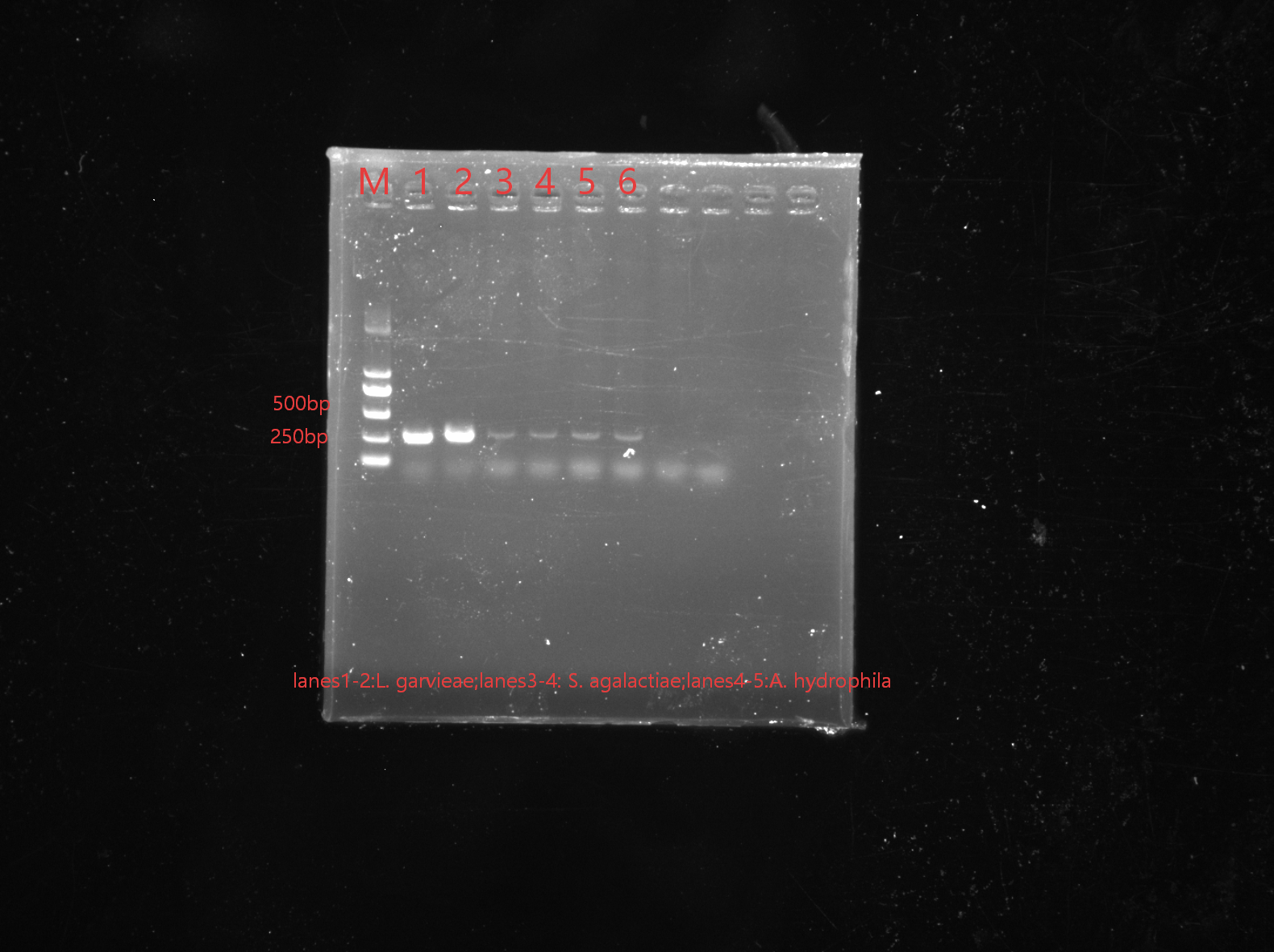

Supplement: Supplementary file 1 — Supplementary Material 1. Supplementary Fig. S1. Specificity assessment of two previously published PCR primer sets for L. garvieae detection. Agarose gel electrophoresis analysis of PCR products amplified from genomic DNA of various bacterial pathogens. (A) PCR using the 16S-23S rRNA ITS-targeting primer set. (B) PCR using the 16S rRNA-targeting primer set. Supplementary Fig. S2. Multiple sequence alignment of the adhE-ywdF target region among fish-derived L. garvieae strains. The aligned sequences include four L. garvieae strains isolated from fish: AP027239.1, AP009333.1, AP009332.1 and AP043994.1. The target region showed 100% nucleotide identity across all tested strains, confirming that the selected target is highly conserved within L. garvieae. Supplementary Fig. S3. Sequence alignment of the target region and RPA-LFD detection of Lactococcus species. (A) Partial nucleotide sequence alignment of the adhE-ywdF target region from L. garvieae and L. petauri. The binding sites of the forward primer RPAF3, the reverse primer RPAR1-bio, and the nfo probe are indicated. (B) RPA-LFD assay results using genomic DNA from L. garvieae, L. petauri, L. lactis, and a no‑template control (NC). Positive signals (test line) were obtained for both L. garvieae and L. petauri, indicating cross‑reactivity. No amplification was observed for L. lactis or the NC. [file 12917_2026_5587_MOESM1_ESM.zip › 12917_2026_5587_MOESM18_ESM.tif]

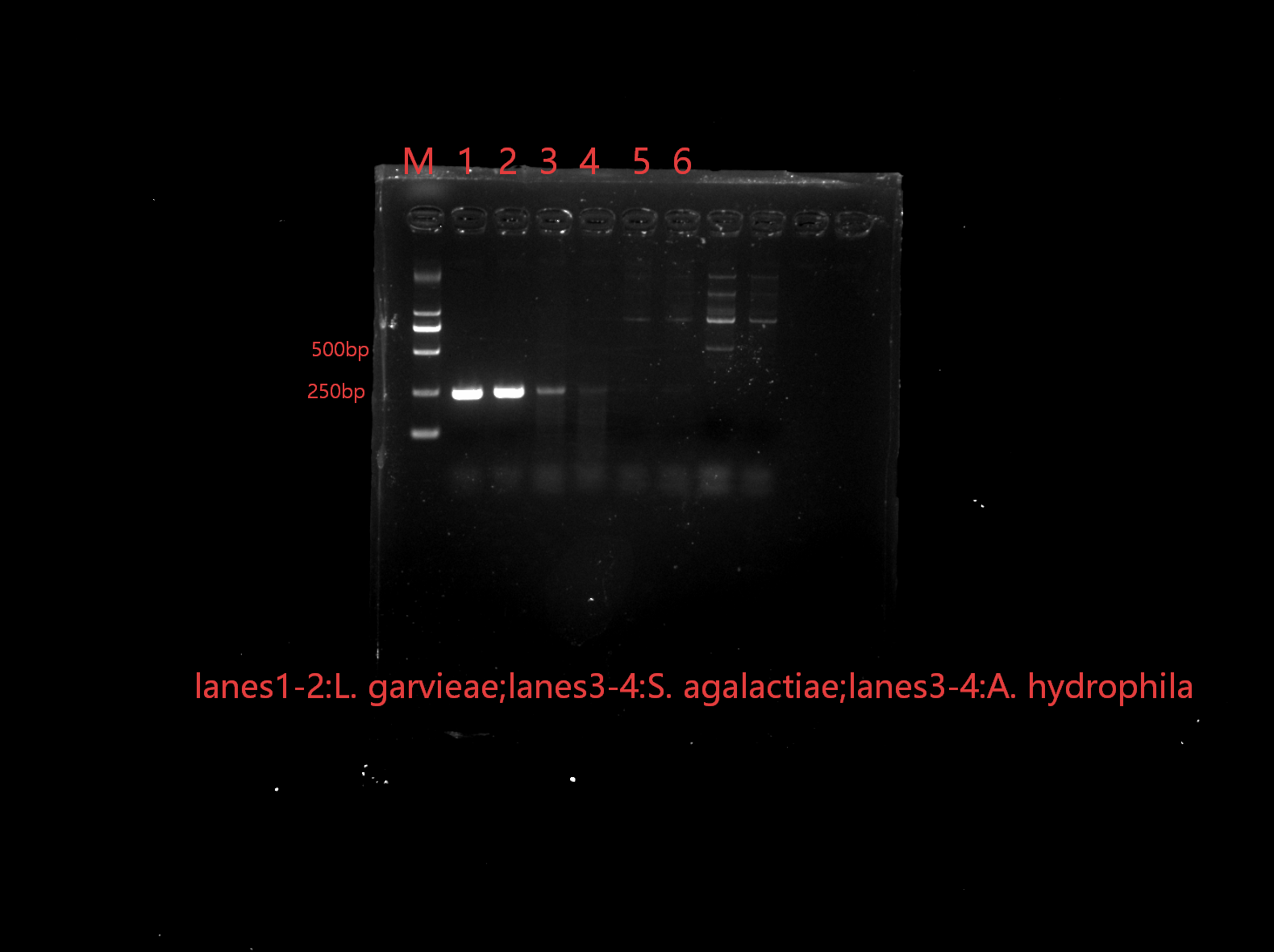

Supplement: Supplementary file 1 — Supplementary Material 1. Supplementary Fig. S1. Specificity assessment of two previously published PCR primer sets for L. garvieae detection. Agarose gel electrophoresis analysis of PCR products amplified from genomic DNA of various bacterial pathogens. (A) PCR using the 16S-23S rRNA ITS-targeting primer set. (B) PCR using the 16S rRNA-targeting primer set. Supplementary Fig. S2. Multiple sequence alignment of the adhE-ywdF target region among fish-derived L. garvieae strains. The aligned sequences include four L. garvieae strains isolated from fish: AP027239.1, AP009333.1, AP009332.1 and AP043994.1. The target region showed 100% nucleotide identity across all tested strains, confirming that the selected target is highly conserved within L. garvieae. Supplementary Fig. S3. Sequence alignment of the target region and RPA-LFD detection of Lactococcus species. (A) Partial nucleotide sequence alignment of the adhE-ywdF target region from L. garvieae and L. petauri. The binding sites of the forward primer RPAF3, the reverse primer RPAR1-bio, and the nfo probe are indicated. (B) RPA-LFD assay results using genomic DNA from L. garvieae, L. petauri, L. lactis, and a no‑template control (NC). Positive signals (test line) were obtained for both L. garvieae and L. petauri, indicating cross‑reactivity. No amplification was observed for L. lactis or the NC. [file 12917_2026_5587_MOESM1_ESM.zip › 12917_2026_5587_MOESM19_ESM.tif]

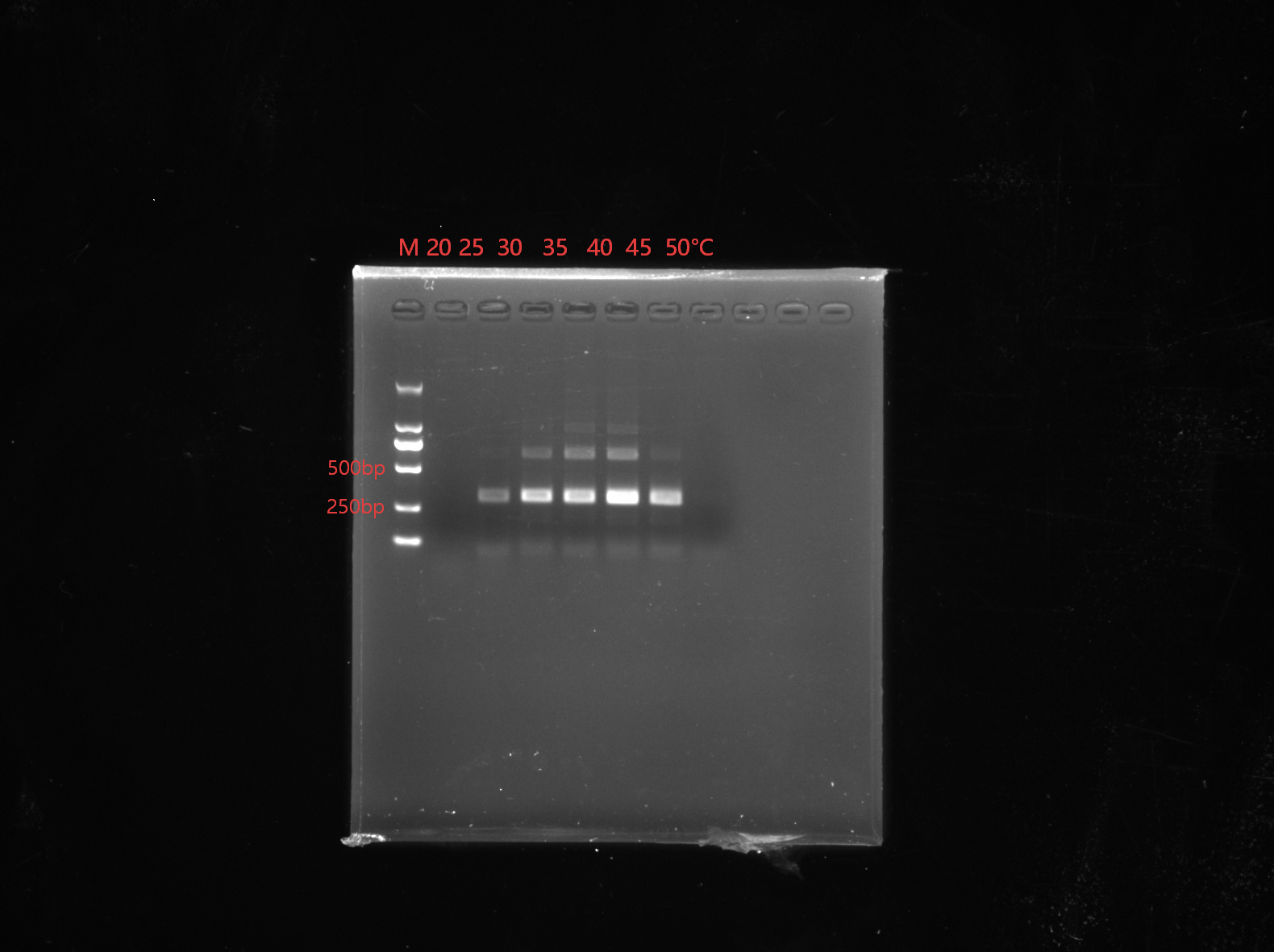

Supplement: Supplementary file 1 — Supplementary Material 1. Supplementary Fig. S1. Specificity assessment of two previously published PCR primer sets for L. garvieae detection. Agarose gel electrophoresis analysis of PCR products amplified from genomic DNA of various bacterial pathogens. (A) PCR using the 16S-23S rRNA ITS-targeting primer set. (B) PCR using the 16S rRNA-targeting primer set. Supplementary Fig. S2. Multiple sequence alignment of the adhE-ywdF target region among fish-derived L. garvieae strains. The aligned sequences include four L. garvieae strains isolated from fish: AP027239.1, AP009333.1, AP009332.1 and AP043994.1. The target region showed 100% nucleotide identity across all tested strains, confirming that the selected target is highly conserved within L. garvieae. Supplementary Fig. S3. Sequence alignment of the target region and RPA-LFD detection of Lactococcus species. (A) Partial nucleotide sequence alignment of the adhE-ywdF target region from L. garvieae and L. petauri. The binding sites of the forward primer RPAF3, the reverse primer RPAR1-bio, and the nfo probe are indicated. (B) RPA-LFD assay results using genomic DNA from L. garvieae, L. petauri, L. lactis, and a no‑template control (NC). Positive signals (test line) were obtained for both L. garvieae and L. petauri, indicating cross‑reactivity. No amplification was observed for L. lactis or the NC. [file 12917_2026_5587_MOESM1_ESM.zip › 12917_2026_5587_MOESM2_ESM.tif]

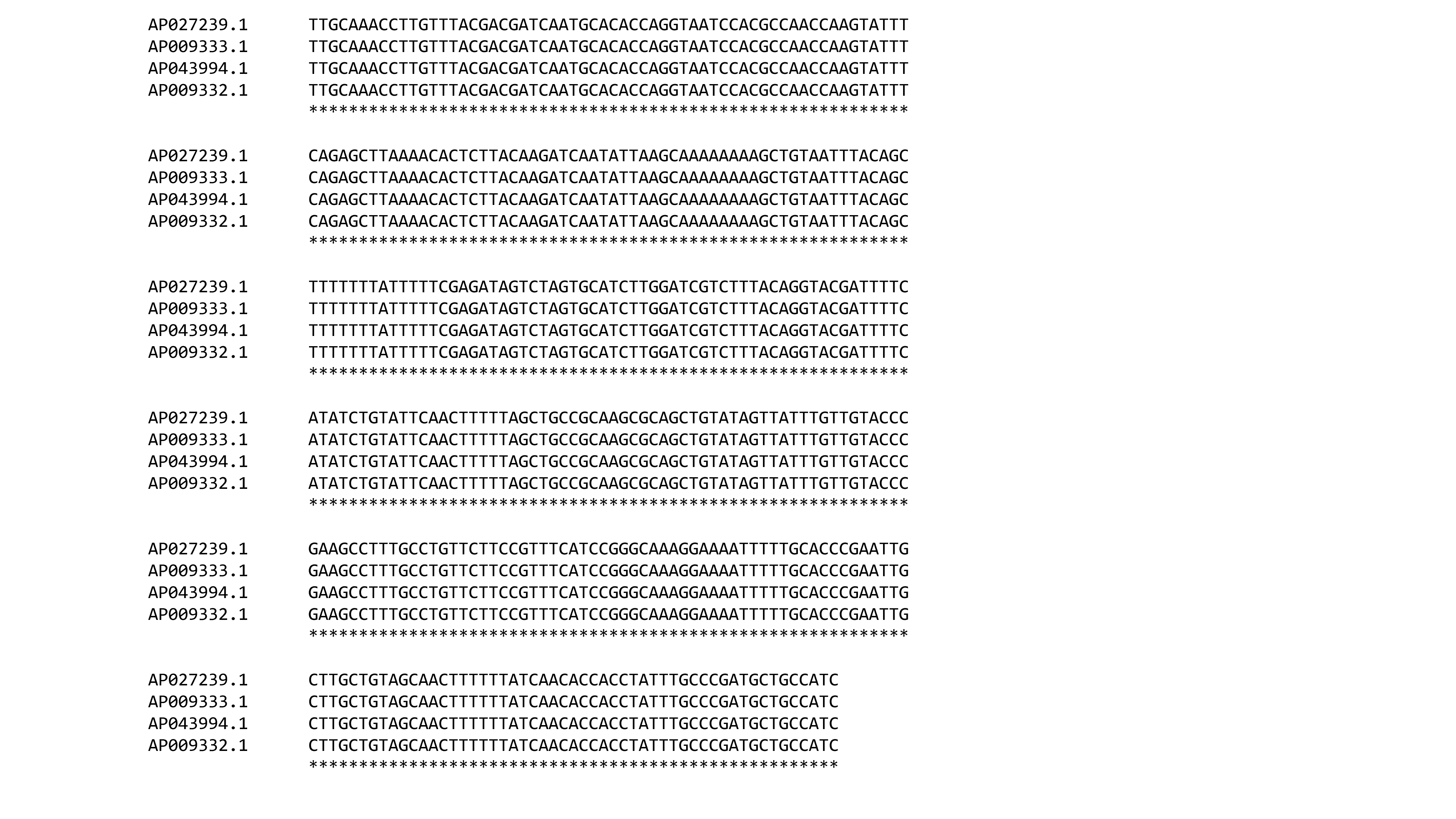

Supplement: Supplementary file 1 — Supplementary Material 1. Supplementary Fig. S1. Specificity assessment of two previously published PCR primer sets for L. garvieae detection. Agarose gel electrophoresis analysis of PCR products amplified from genomic DNA of various bacterial pathogens. (A) PCR using the 16S-23S rRNA ITS-targeting primer set. (B) PCR using the 16S rRNA-targeting primer set. Supplementary Fig. S2. Multiple sequence alignment of the adhE-ywdF target region among fish-derived L. garvieae strains. The aligned sequences include four L. garvieae strains isolated from fish: AP027239.1, AP009333.1, AP009332.1 and AP043994.1. The target region showed 100% nucleotide identity across all tested strains, confirming that the selected target is highly conserved within L. garvieae. Supplementary Fig. S3. Sequence alignment of the target region and RPA-LFD detection of Lactococcus species. (A) Partial nucleotide sequence alignment of the adhE-ywdF target region from L. garvieae and L. petauri. The binding sites of the forward primer RPAF3, the reverse primer RPAR1-bio, and the nfo probe are indicated. (B) RPA-LFD assay results using genomic DNA from L. garvieae, L. petauri, L. lactis, and a no‑template control (NC). Positive signals (test line) were obtained for both L. garvieae and L. petauri, indicating cross‑reactivity. No amplification was observed for L. lactis or the NC. [file 12917_2026_5587_MOESM1_ESM.zip › 12917_2026_5587_MOESM20_ESM.tif]

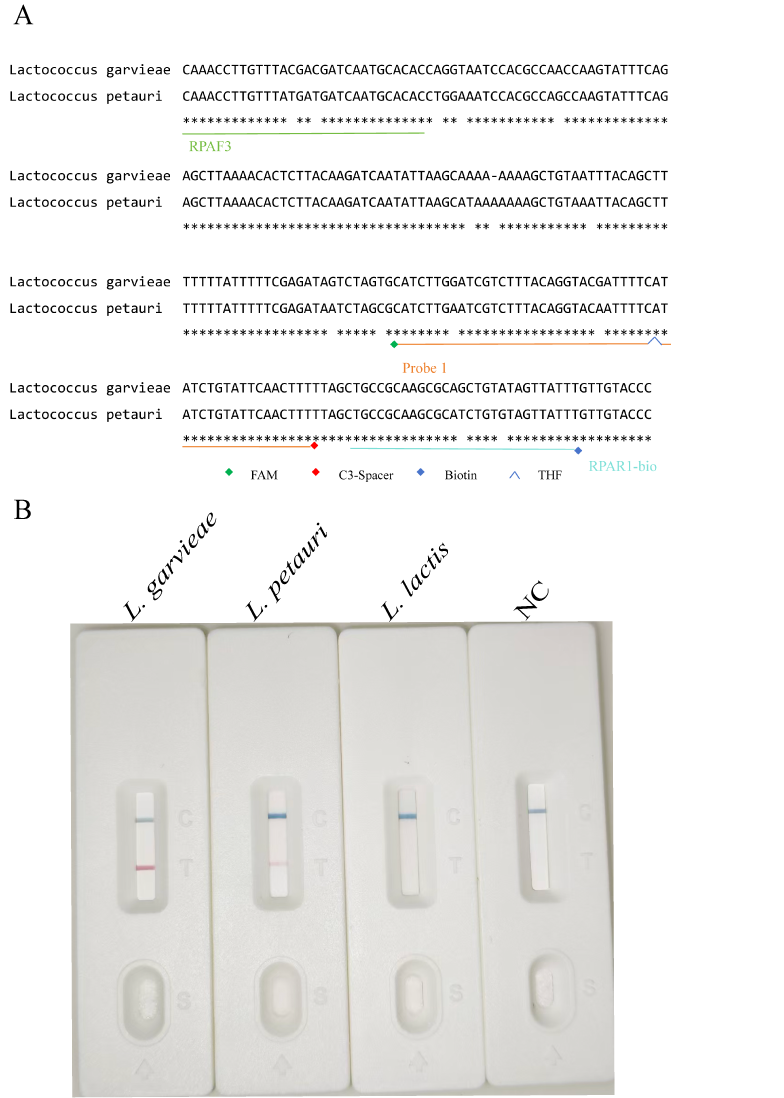

Supplement: Supplementary file 1 — Supplementary Material 1. Supplementary Fig. S1. Specificity assessment of two previously published PCR primer sets for L. garvieae detection. Agarose gel electrophoresis analysis of PCR products amplified from genomic DNA of various bacterial pathogens. (A) PCR using the 16S-23S rRNA ITS-targeting primer set. (B) PCR using the 16S rRNA-targeting primer set. Supplementary Fig. S2. Multiple sequence alignment of the adhE-ywdF target region among fish-derived L. garvieae strains. The aligned sequences include four L. garvieae strains isolated from fish: AP027239.1, AP009333.1, AP009332.1 and AP043994.1. The target region showed 100% nucleotide identity across all tested strains, confirming that the selected target is highly conserved within L. garvieae. Supplementary Fig. S3. Sequence alignment of the target region and RPA-LFD detection of Lactococcus species. (A) Partial nucleotide sequence alignment of the adhE-ywdF target region from L. garvieae and L. petauri. The binding sites of the forward primer RPAF3, the reverse primer RPAR1-bio, and the nfo probe are indicated. (B) RPA-LFD assay results using genomic DNA from L. garvieae, L. petauri, L. lactis, and a no‑template control (NC). Positive signals (test line) were obtained for both L. garvieae and L. petauri, indicating cross‑reactivity. No amplification was observed for L. lactis or the NC. [file 12917_2026_5587_MOESM1_ESM.zip › 12917_2026_5587_MOESM21_ESM.tif]

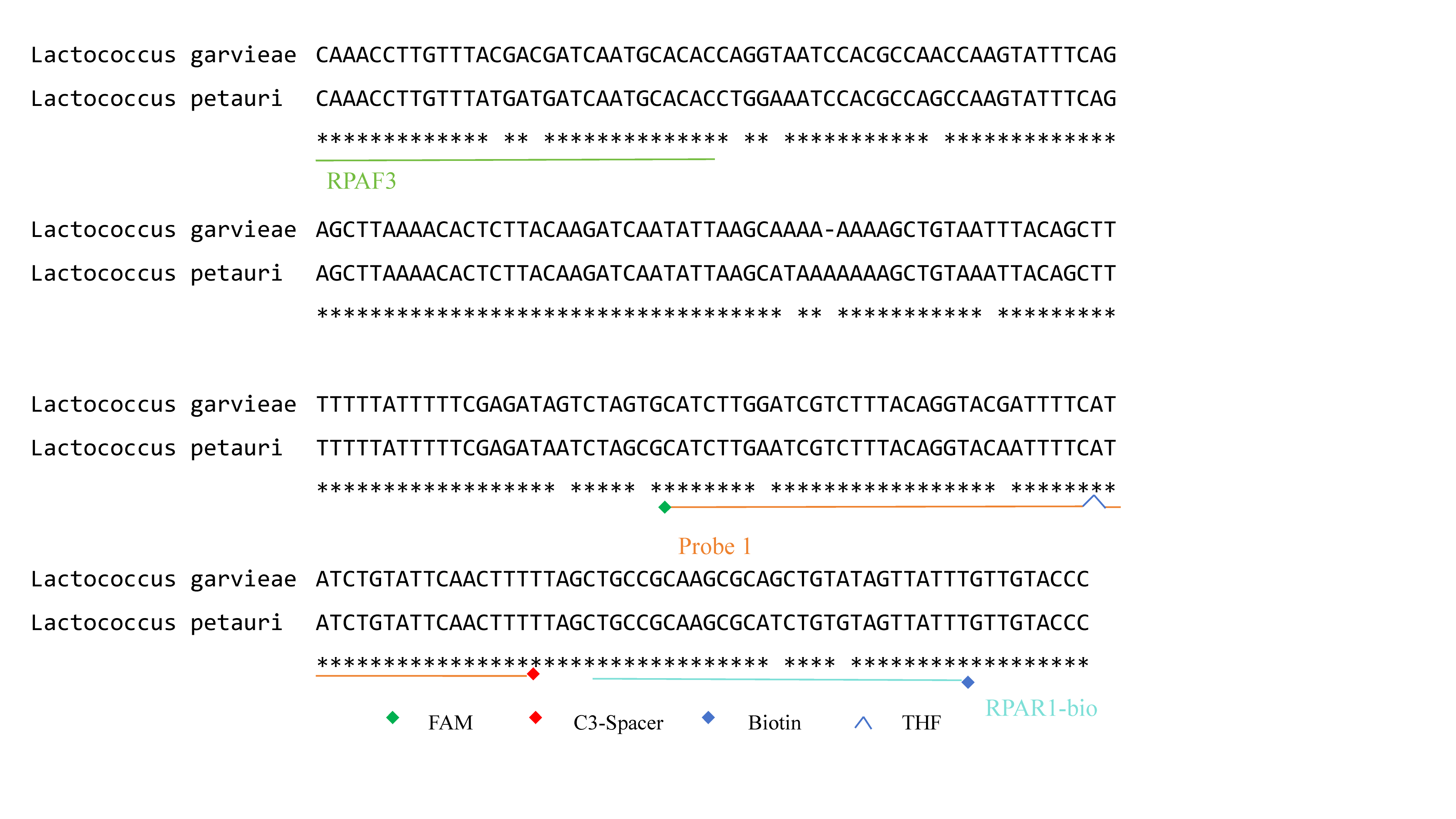

Supplement: Supplementary file 1 — Supplementary Material 1. Supplementary Fig. S1. Specificity assessment of two previously published PCR primer sets for L. garvieae detection. Agarose gel electrophoresis analysis of PCR products amplified from genomic DNA of various bacterial pathogens. (A) PCR using the 16S-23S rRNA ITS-targeting primer set. (B) PCR using the 16S rRNA-targeting primer set. Supplementary Fig. S2. Multiple sequence alignment of the adhE-ywdF target region among fish-derived L. garvieae strains. The aligned sequences include four L. garvieae strains isolated from fish: AP027239.1, AP009333.1, AP009332.1 and AP043994.1. The target region showed 100% nucleotide identity across all tested strains, confirming that the selected target is highly conserved within L. garvieae. Supplementary Fig. S3. Sequence alignment of the target region and RPA-LFD detection of Lactococcus species. (A) Partial nucleotide sequence alignment of the adhE-ywdF target region from L. garvieae and L. petauri. The binding sites of the forward primer RPAF3, the reverse primer RPAR1-bio, and the nfo probe are indicated. (B) RPA-LFD assay results using genomic DNA from L. garvieae, L. petauri, L. lactis, and a no‑template control (NC). Positive signals (test line) were obtained for both L. garvieae and L. petauri, indicating cross‑reactivity. No amplification was observed for L. lactis or the NC. [file 12917_2026_5587_MOESM1_ESM.zip › 12917_2026_5587_MOESM22_ESM.tif]

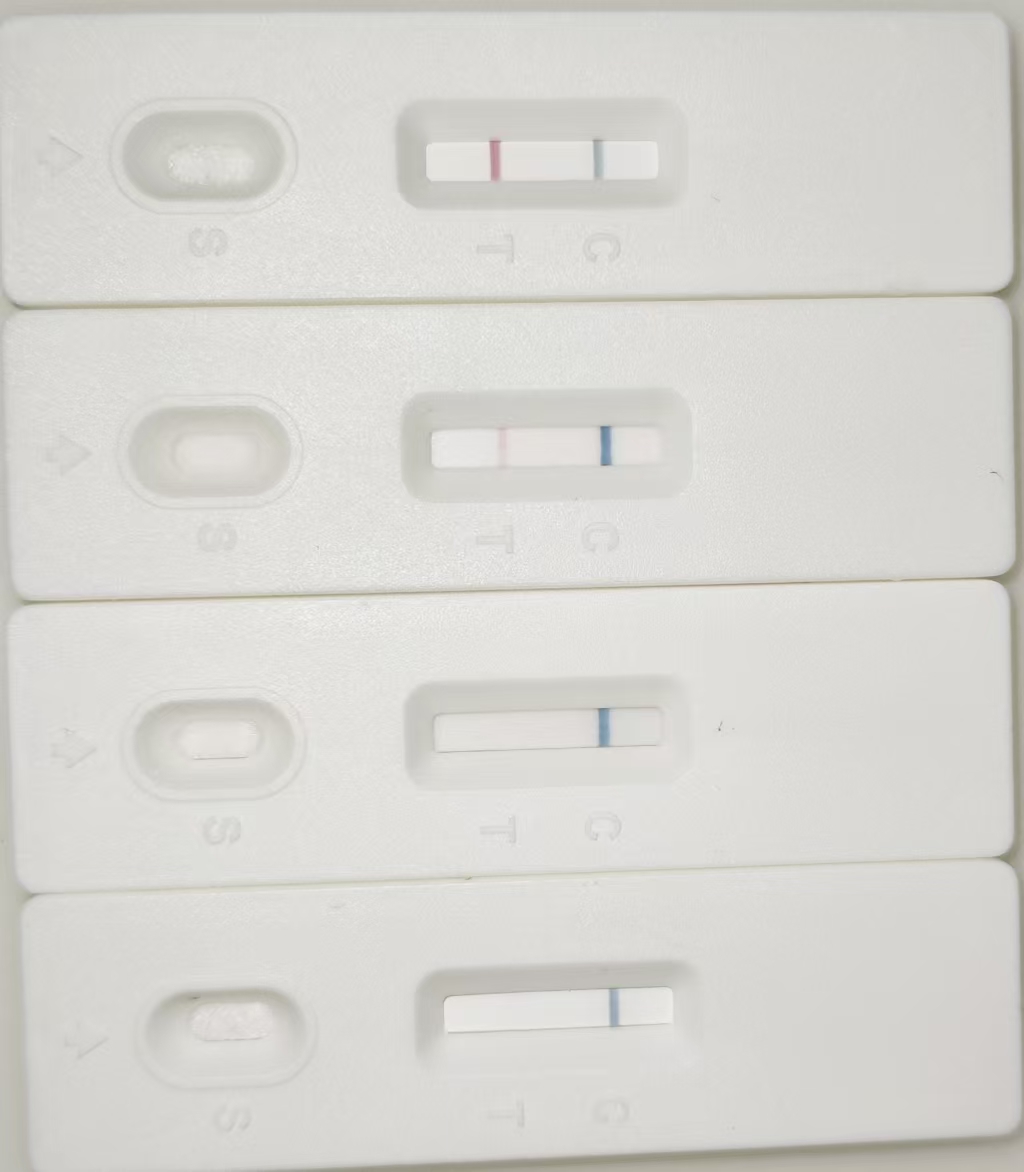

Supplement: Supplementary file 1 — Supplementary Material 1. Supplementary Fig. S1. Specificity assessment of two previously published PCR primer sets for L. garvieae detection. Agarose gel electrophoresis analysis of PCR products amplified from genomic DNA of various bacterial pathogens. (A) PCR using the 16S-23S rRNA ITS-targeting primer set. (B) PCR using the 16S rRNA-targeting primer set. Supplementary Fig. S2. Multiple sequence alignment of the adhE-ywdF target region among fish-derived L. garvieae strains. The aligned sequences include four L. garvieae strains isolated from fish: AP027239.1, AP009333.1, AP009332.1 and AP043994.1. The target region showed 100% nucleotide identity across all tested strains, confirming that the selected target is highly conserved within L. garvieae. Supplementary Fig. S3. Sequence alignment of the target region and RPA-LFD detection of Lactococcus species. (A) Partial nucleotide sequence alignment of the adhE-ywdF target region from L. garvieae and L. petauri. The binding sites of the forward primer RPAF3, the reverse primer RPAR1-bio, and the nfo probe are indicated. (B) RPA-LFD assay results using genomic DNA from L. garvieae, L. petauri, L. lactis, and a no‑template control (NC). Positive signals (test line) were obtained for both L. garvieae and L. petauri, indicating cross‑reactivity. No amplification was observed for L. lactis or the NC. [file 12917_2026_5587_MOESM1_ESM.zip › 12917_2026_5587_MOESM23_ESM.jpg]

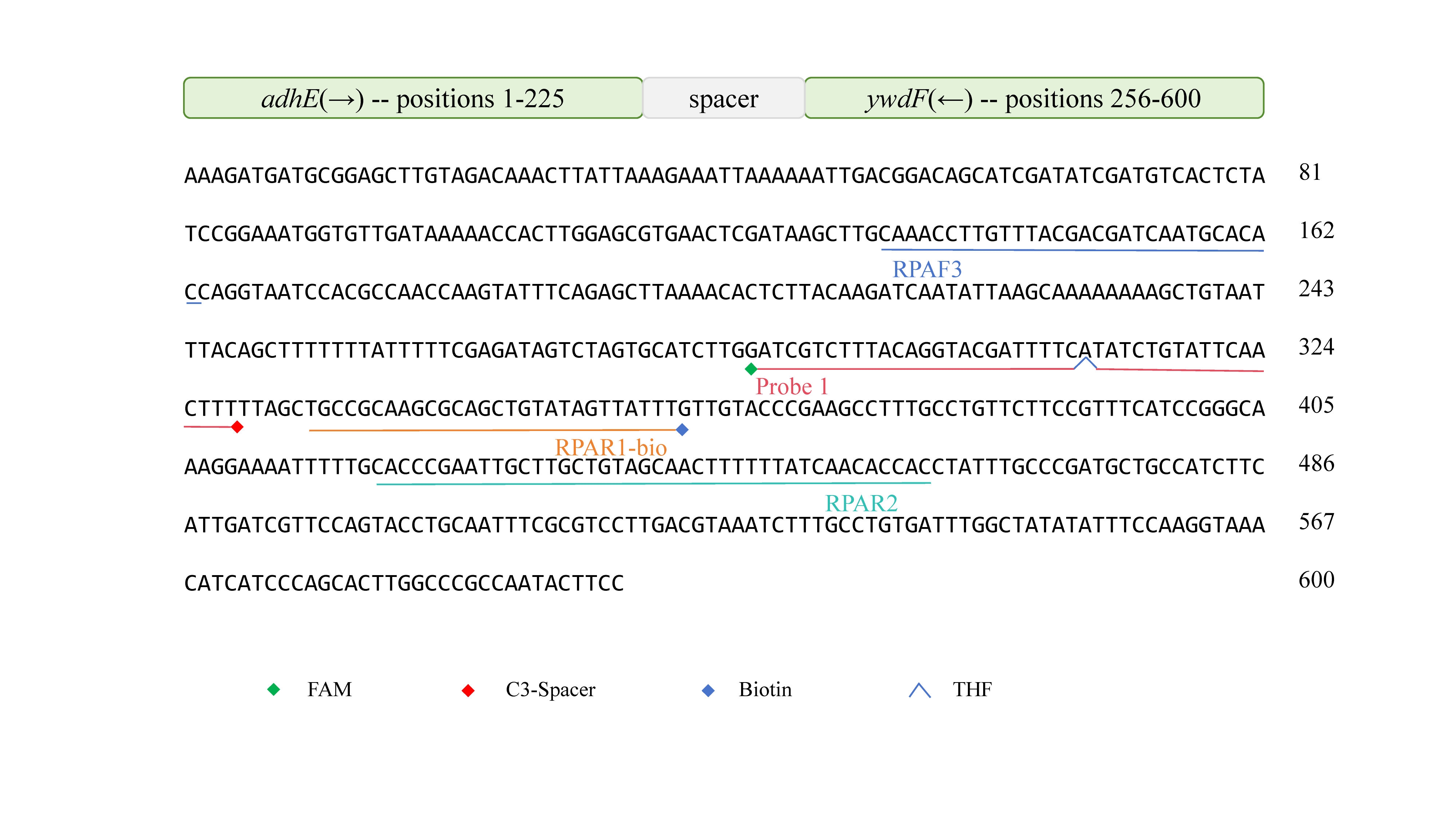

Supplement: Supplementary file 1 — Supplementary Material 1. Supplementary Fig. S1. Specificity assessment of two previously published PCR primer sets for L. garvieae detection. Agarose gel electrophoresis analysis of PCR products amplified from genomic DNA of various bacterial pathogens. (A) PCR using the 16S-23S rRNA ITS-targeting primer set. (B) PCR using the 16S rRNA-targeting primer set. Supplementary Fig. S2. Multiple sequence alignment of the adhE-ywdF target region among fish-derived L. garvieae strains. The aligned sequences include four L. garvieae strains isolated from fish: AP027239.1, AP009333.1, AP009332.1 and AP043994.1. The target region showed 100% nucleotide identity across all tested strains, confirming that the selected target is highly conserved within L. garvieae. Supplementary Fig. S3. Sequence alignment of the target region and RPA-LFD detection of Lactococcus species. (A) Partial nucleotide sequence alignment of the adhE-ywdF target region from L. garvieae and L. petauri. The binding sites of the forward primer RPAF3, the reverse primer RPAR1-bio, and the nfo probe are indicated. (B) RPA-LFD assay results using genomic DNA from L. garvieae, L. petauri, L. lactis, and a no‑template control (NC). Positive signals (test line) were obtained for both L. garvieae and L. petauri, indicating cross‑reactivity. No amplification was observed for L. lactis or the NC. [file 12917_2026_5587_MOESM1_ESM.zip › 12917_2026_5587_MOESM24_ESM.tif]

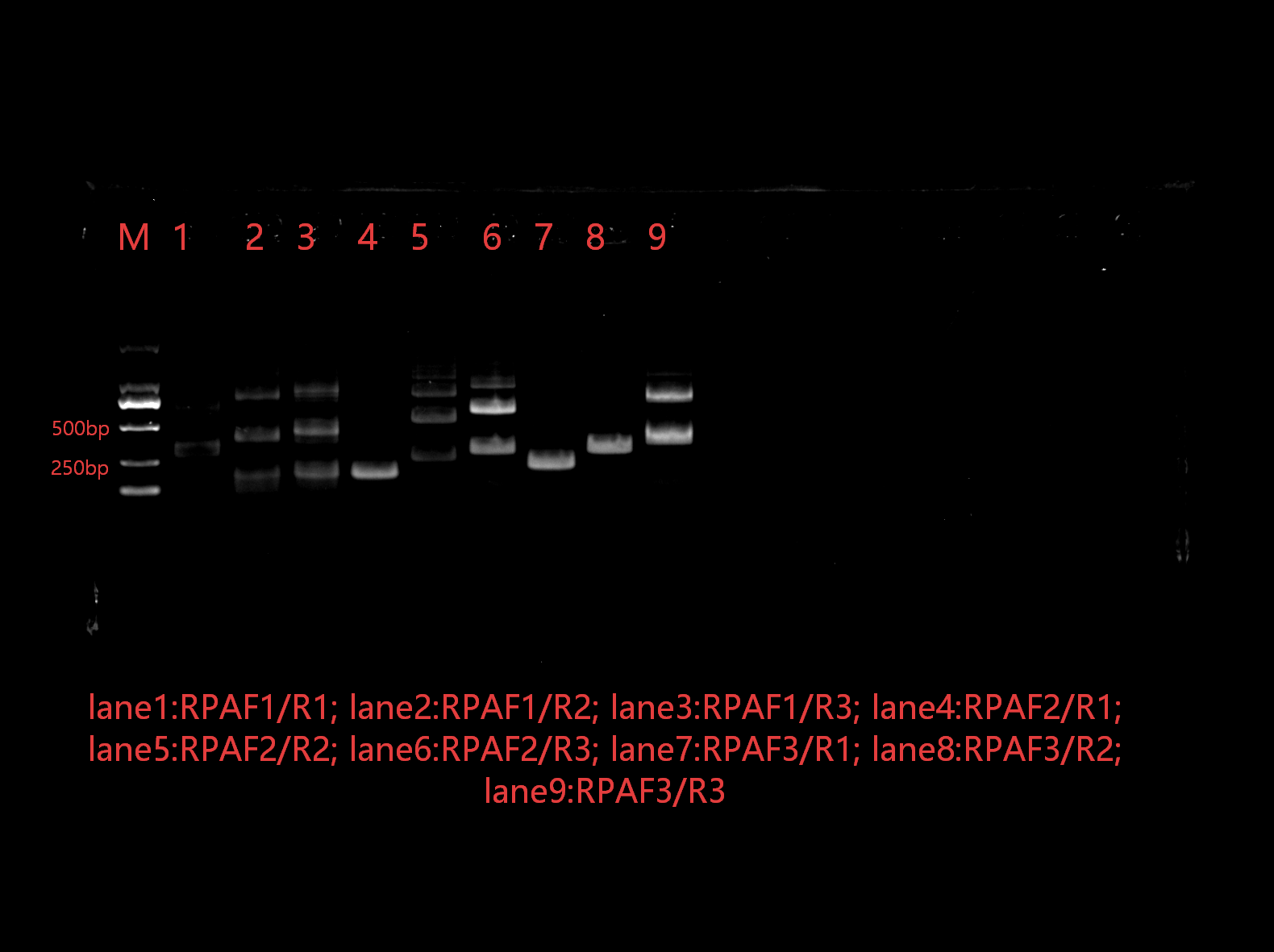

Supplement: Supplementary file 1 — Supplementary Material 1. Supplementary Fig. S1. Specificity assessment of two previously published PCR primer sets for L. garvieae detection. Agarose gel electrophoresis analysis of PCR products amplified from genomic DNA of various bacterial pathogens. (A) PCR using the 16S-23S rRNA ITS-targeting primer set. (B) PCR using the 16S rRNA-targeting primer set. Supplementary Fig. S2. Multiple sequence alignment of the adhE-ywdF target region among fish-derived L. garvieae strains. The aligned sequences include four L. garvieae strains isolated from fish: AP027239.1, AP009333.1, AP009332.1 and AP043994.1. The target region showed 100% nucleotide identity across all tested strains, confirming that the selected target is highly conserved within L. garvieae. Supplementary Fig. S3. Sequence alignment of the target region and RPA-LFD detection of Lactococcus species. (A) Partial nucleotide sequence alignment of the adhE-ywdF target region from L. garvieae and L. petauri. The binding sites of the forward primer RPAF3, the reverse primer RPAR1-bio, and the nfo probe are indicated. (B) RPA-LFD assay results using genomic DNA from L. garvieae, L. petauri, L. lactis, and a no‑template control (NC). Positive signals (test line) were obtained for both L. garvieae and L. petauri, indicating cross‑reactivity. No amplification was observed for L. lactis or the NC. [file 12917_2026_5587_MOESM1_ESM.zip › 12917_2026_5587_MOESM25_ESM.tif]

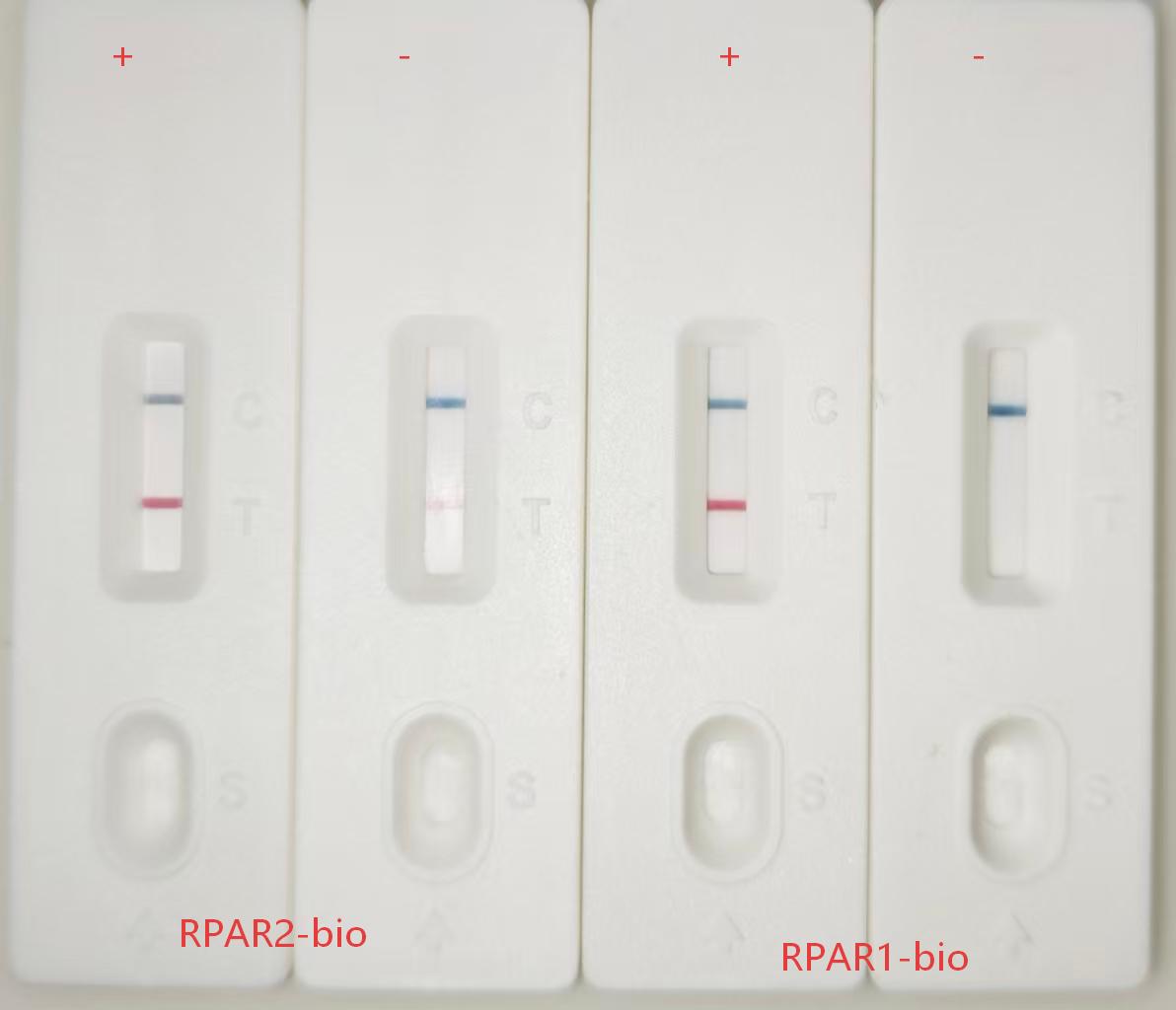

Supplement: Supplementary file 1 — Supplementary Material 1. Supplementary Fig. S1. Specificity assessment of two previously published PCR primer sets for L. garvieae detection. Agarose gel electrophoresis analysis of PCR products amplified from genomic DNA of various bacterial pathogens. (A) PCR using the 16S-23S rRNA ITS-targeting primer set. (B) PCR using the 16S rRNA-targeting primer set. Supplementary Fig. S2. Multiple sequence alignment of the adhE-ywdF target region among fish-derived L. garvieae strains. The aligned sequences include four L. garvieae strains isolated from fish: AP027239.1, AP009333.1, AP009332.1 and AP043994.1. The target region showed 100% nucleotide identity across all tested strains, confirming that the selected target is highly conserved within L. garvieae. Supplementary Fig. S3. Sequence alignment of the target region and RPA-LFD detection of Lactococcus species. (A) Partial nucleotide sequence alignment of the adhE-ywdF target region from L. garvieae and L. petauri. The binding sites of the forward primer RPAF3, the reverse primer RPAR1-bio, and the nfo probe are indicated. (B) RPA-LFD assay results using genomic DNA from L. garvieae, L. petauri, L. lactis, and a no‑template control (NC). Positive signals (test line) were obtained for both L. garvieae and L. petauri, indicating cross‑reactivity. No amplification was observed for L. lactis or the NC. [file 12917_2026_5587_MOESM1_ESM.zip › 12917_2026_5587_MOESM26_ESM.jpg]

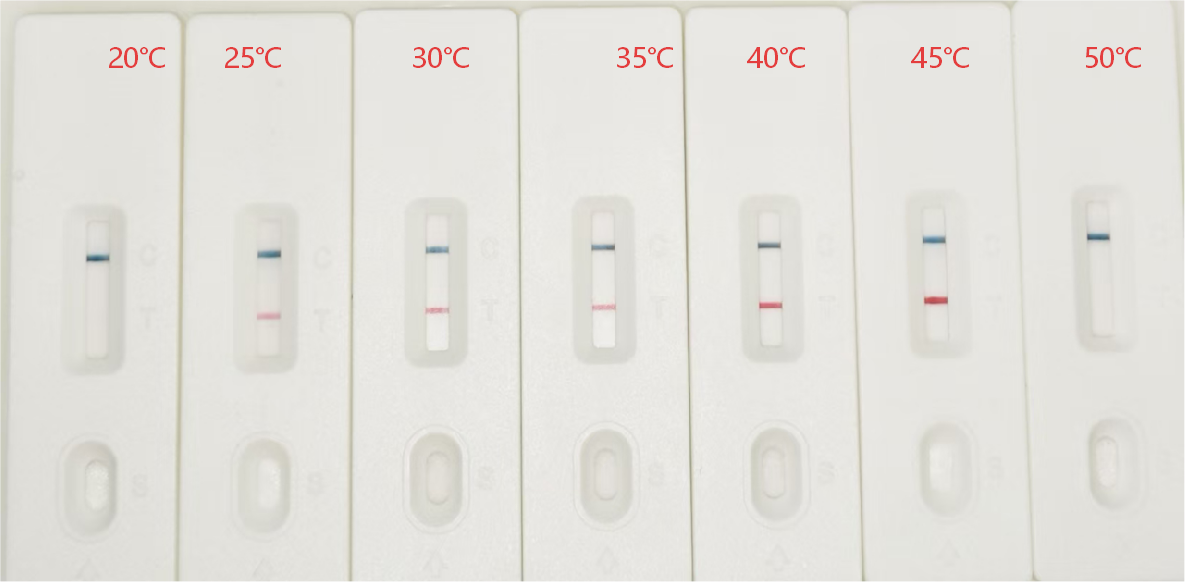

Supplement: Supplementary file 1 — Supplementary Material 1. Supplementary Fig. S1. Specificity assessment of two previously published PCR primer sets for L. garvieae detection. Agarose gel electrophoresis analysis of PCR products amplified from genomic DNA of various bacterial pathogens. (A) PCR using the 16S-23S rRNA ITS-targeting primer set. (B) PCR using the 16S rRNA-targeting primer set. Supplementary Fig. S2. Multiple sequence alignment of the adhE-ywdF target region among fish-derived L. garvieae strains. The aligned sequences include four L. garvieae strains isolated from fish: AP027239.1, AP009333.1, AP009332.1 and AP043994.1. The target region showed 100% nucleotide identity across all tested strains, confirming that the selected target is highly conserved within L. garvieae. Supplementary Fig. S3. Sequence alignment of the target region and RPA-LFD detection of Lactococcus species. (A) Partial nucleotide sequence alignment of the adhE-ywdF target region from L. garvieae and L. petauri. The binding sites of the forward primer RPAF3, the reverse primer RPAR1-bio, and the nfo probe are indicated. (B) RPA-LFD assay results using genomic DNA from L. garvieae, L. petauri, L. lactis, and a no‑template control (NC). Positive signals (test line) were obtained for both L. garvieae and L. petauri, indicating cross‑reactivity. No amplification was observed for L. lactis or the NC. [file 12917_2026_5587_MOESM1_ESM.zip › 12917_2026_5587_MOESM3_ESM.png]

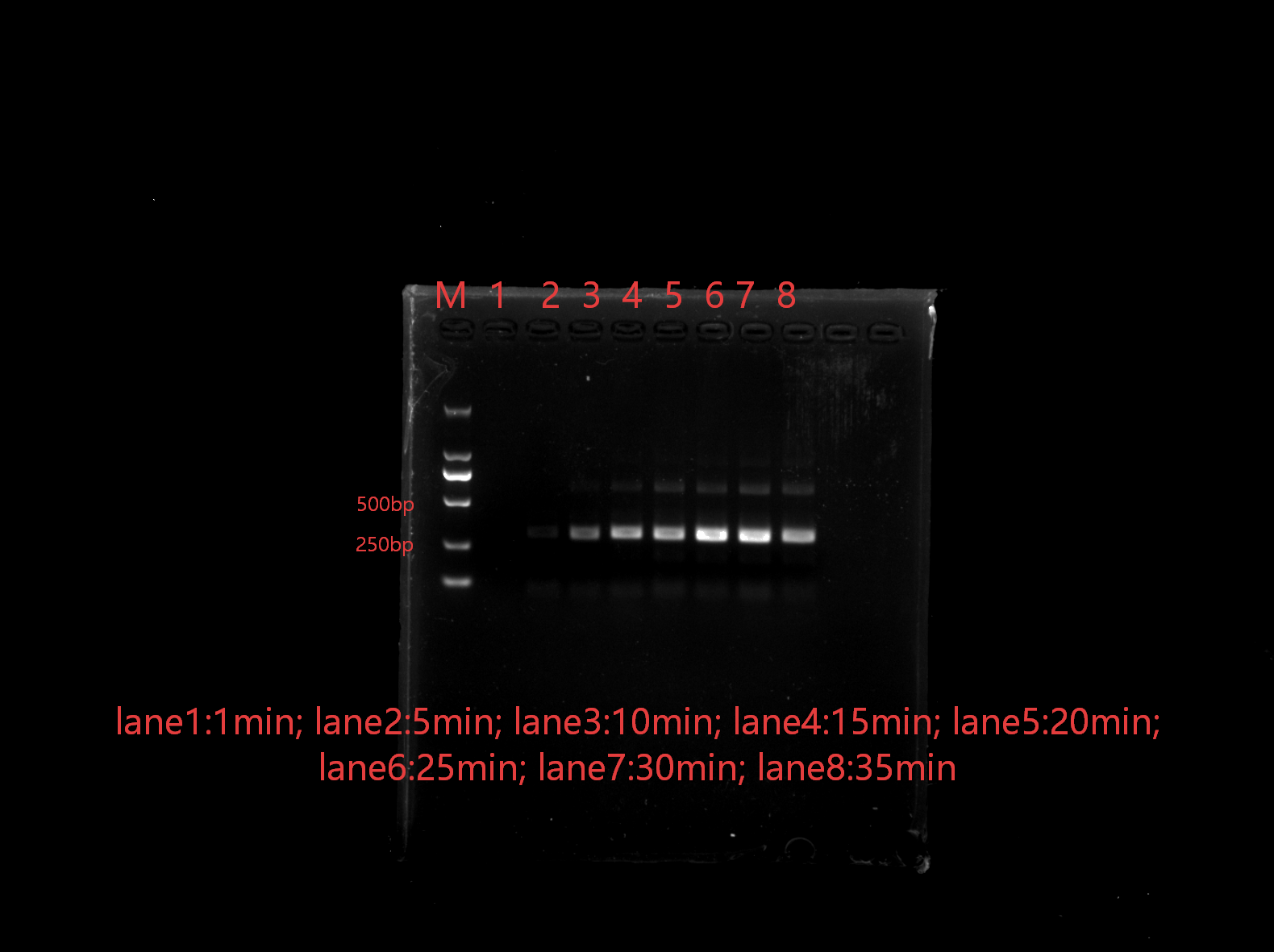

Supplement: Supplementary file 1 — Supplementary Material 1. Supplementary Fig. S1. Specificity assessment of two previously published PCR primer sets for L. garvieae detection. Agarose gel electrophoresis analysis of PCR products amplified from genomic DNA of various bacterial pathogens. (A) PCR using the 16S-23S rRNA ITS-targeting primer set. (B) PCR using the 16S rRNA-targeting primer set. Supplementary Fig. S2. Multiple sequence alignment of the adhE-ywdF target region among fish-derived L. garvieae strains. The aligned sequences include four L. garvieae strains isolated from fish: AP027239.1, AP009333.1, AP009332.1 and AP043994.1. The target region showed 100% nucleotide identity across all tested strains, confirming that the selected target is highly conserved within L. garvieae. Supplementary Fig. S3. Sequence alignment of the target region and RPA-LFD detection of Lactococcus species. (A) Partial nucleotide sequence alignment of the adhE-ywdF target region from L. garvieae and L. petauri. The binding sites of the forward primer RPAF3, the reverse primer RPAR1-bio, and the nfo probe are indicated. (B) RPA-LFD assay results using genomic DNA from L. garvieae, L. petauri, L. lactis, and a no‑template control (NC). Positive signals (test line) were obtained for both L. garvieae and L. petauri, indicating cross‑reactivity. No amplification was observed for L. lactis or the NC. [file 12917_2026_5587_MOESM1_ESM.zip › 12917_2026_5587_MOESM4_ESM.tif]

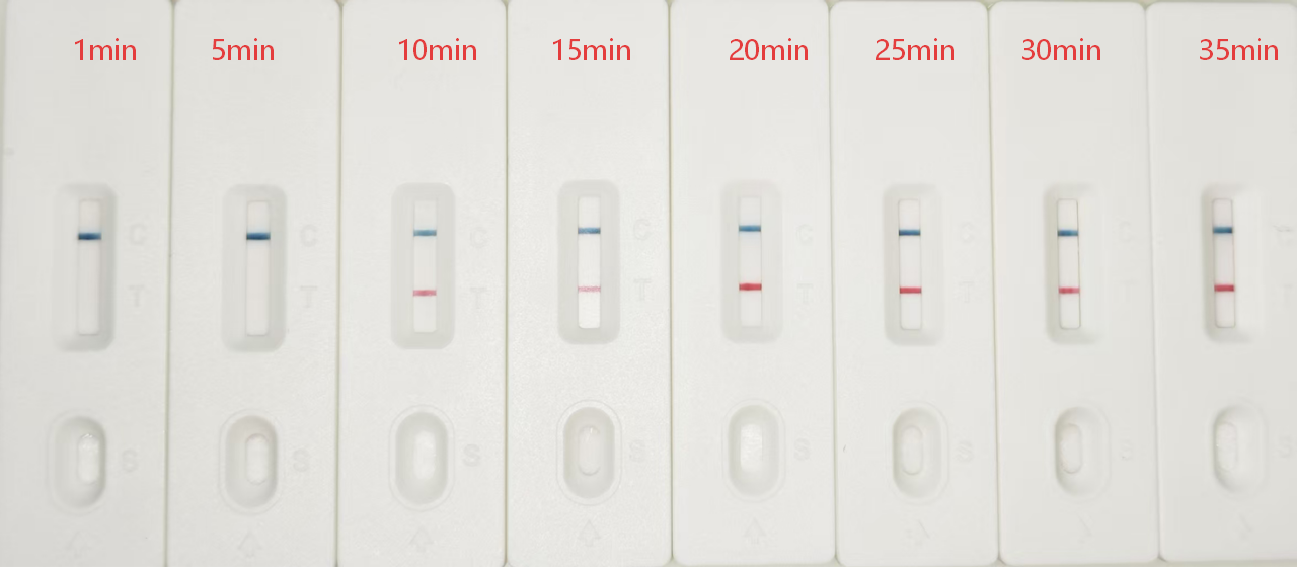

Supplement: Supplementary file 1 — Supplementary Material 1. Supplementary Fig. S1. Specificity assessment of two previously published PCR primer sets for L. garvieae detection. Agarose gel electrophoresis analysis of PCR products amplified from genomic DNA of various bacterial pathogens. (A) PCR using the 16S-23S rRNA ITS-targeting primer set. (B) PCR using the 16S rRNA-targeting primer set. Supplementary Fig. S2. Multiple sequence alignment of the adhE-ywdF target region among fish-derived L. garvieae strains. The aligned sequences include four L. garvieae strains isolated from fish: AP027239.1, AP009333.1, AP009332.1 and AP043994.1. The target region showed 100% nucleotide identity across all tested strains, confirming that the selected target is highly conserved within L. garvieae. Supplementary Fig. S3. Sequence alignment of the target region and RPA-LFD detection of Lactococcus species. (A) Partial nucleotide sequence alignment of the adhE-ywdF target region from L. garvieae and L. petauri. The binding sites of the forward primer RPAF3, the reverse primer RPAR1-bio, and the nfo probe are indicated. (B) RPA-LFD assay results using genomic DNA from L. garvieae, L. petauri, L. lactis, and a no‑template control (NC). Positive signals (test line) were obtained for both L. garvieae and L. petauri, indicating cross‑reactivity. No amplification was observed for L. lactis or the NC. [file 12917_2026_5587_MOESM1_ESM.zip › 12917_2026_5587_MOESM5_ESM.png]

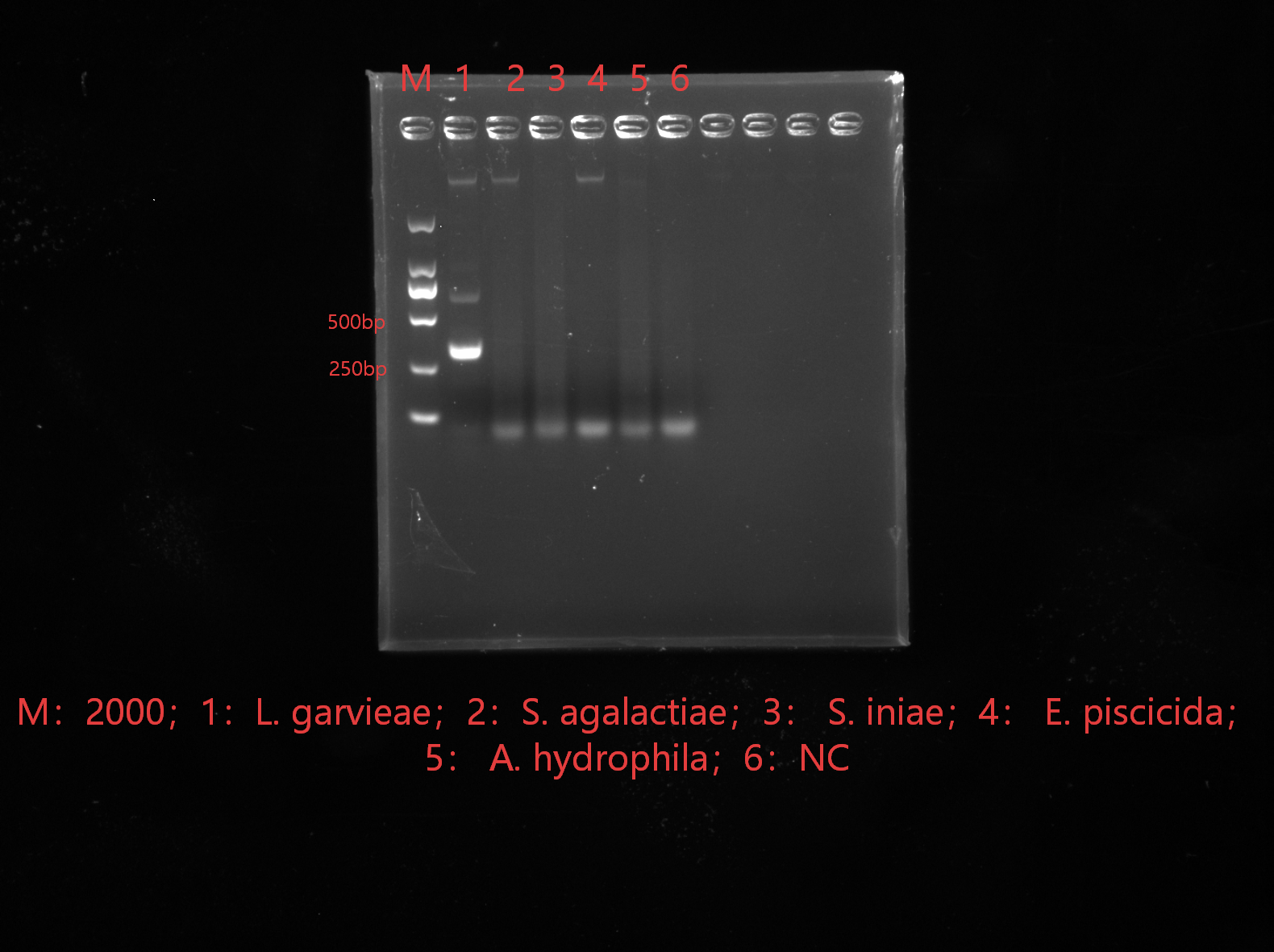

Supplement: Supplementary file 1 — Supplementary Material 1. Supplementary Fig. S1. Specificity assessment of two previously published PCR primer sets for L. garvieae detection. Agarose gel electrophoresis analysis of PCR products amplified from genomic DNA of various bacterial pathogens. (A) PCR using the 16S-23S rRNA ITS-targeting primer set. (B) PCR using the 16S rRNA-targeting primer set. Supplementary Fig. S2. Multiple sequence alignment of the adhE-ywdF target region among fish-derived L. garvieae strains. The aligned sequences include four L. garvieae strains isolated from fish: AP027239.1, AP009333.1, AP009332.1 and AP043994.1. The target region showed 100% nucleotide identity across all tested strains, confirming that the selected target is highly conserved within L. garvieae. Supplementary Fig. S3. Sequence alignment of the target region and RPA-LFD detection of Lactococcus species. (A) Partial nucleotide sequence alignment of the adhE-ywdF target region from L. garvieae and L. petauri. The binding sites of the forward primer RPAF3, the reverse primer RPAR1-bio, and the nfo probe are indicated. (B) RPA-LFD assay results using genomic DNA from L. garvieae, L. petauri, L. lactis, and a no‑template control (NC). Positive signals (test line) were obtained for both L. garvieae and L. petauri, indicating cross‑reactivity. No amplification was observed for L. lactis or the NC. [file 12917_2026_5587_MOESM1_ESM.zip › 12917_2026_5587_MOESM6_ESM.tif]

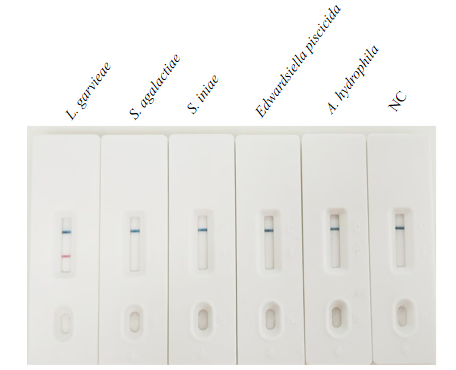

Supplement: Supplementary file 1 — Supplementary Material 1. Supplementary Fig. S1. Specificity assessment of two previously published PCR primer sets for L. garvieae detection. Agarose gel electrophoresis analysis of PCR products amplified from genomic DNA of various bacterial pathogens. (A) PCR using the 16S-23S rRNA ITS-targeting primer set. (B) PCR using the 16S rRNA-targeting primer set. Supplementary Fig. S2. Multiple sequence alignment of the adhE-ywdF target region among fish-derived L. garvieae strains. The aligned sequences include four L. garvieae strains isolated from fish: AP027239.1, AP009333.1, AP009332.1 and AP043994.1. The target region showed 100% nucleotide identity across all tested strains, confirming that the selected target is highly conserved within L. garvieae. Supplementary Fig. S3. Sequence alignment of the target region and RPA-LFD detection of Lactococcus species. (A) Partial nucleotide sequence alignment of the adhE-ywdF target region from L. garvieae and L. petauri. The binding sites of the forward primer RPAF3, the reverse primer RPAR1-bio, and the nfo probe are indicated. (B) RPA-LFD assay results using genomic DNA from L. garvieae, L. petauri, L. lactis, and a no‑template control (NC). Positive signals (test line) were obtained for both L. garvieae and L. petauri, indicating cross‑reactivity. No amplification was observed for L. lactis or the NC. [file 12917_2026_5587_MOESM1_ESM.zip › 12917_2026_5587_MOESM7_ESM.png]

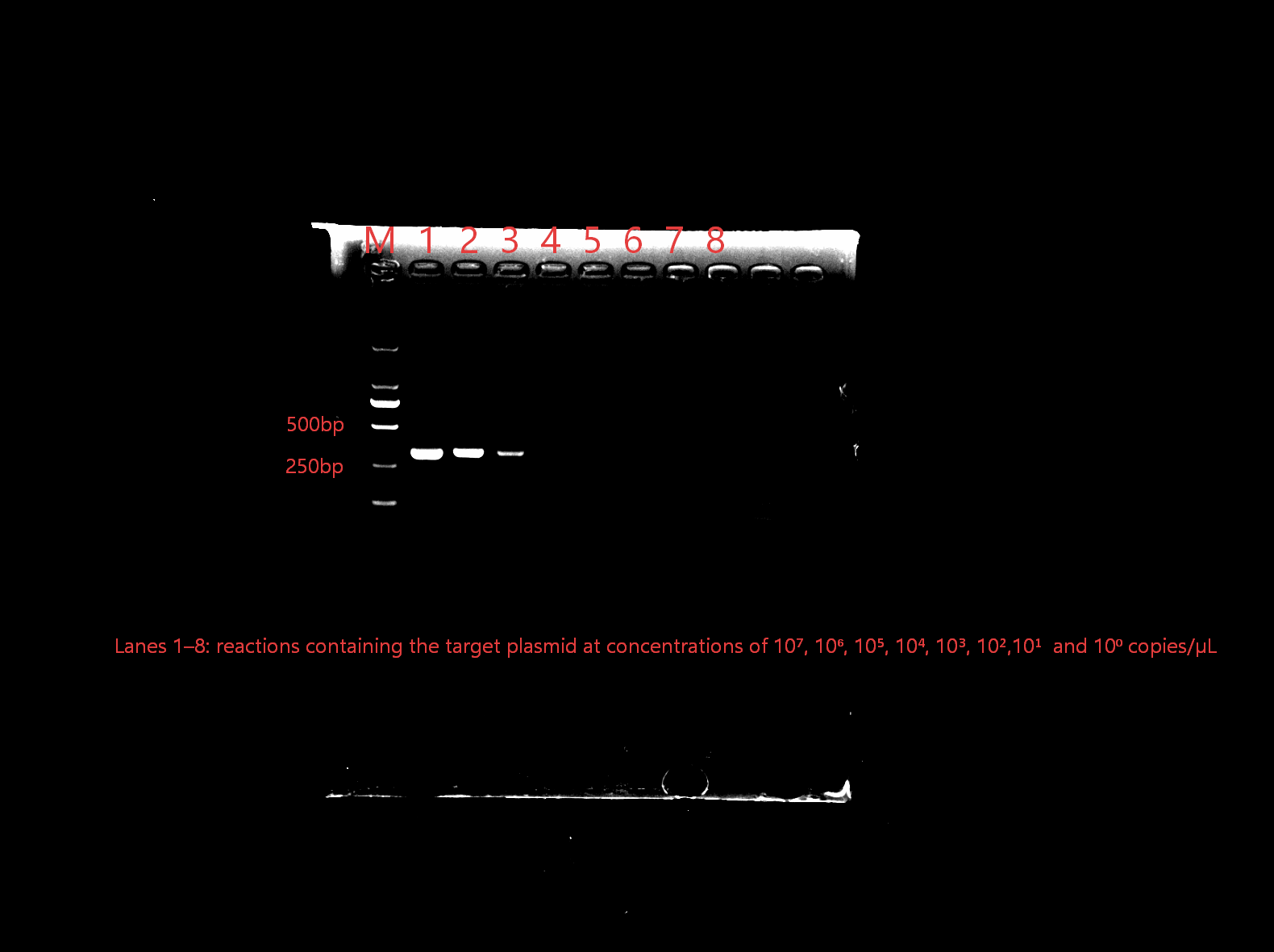

Supplement: Supplementary file 1 — Supplementary Material 1. Supplementary Fig. S1. Specificity assessment of two previously published PCR primer sets for L. garvieae detection. Agarose gel electrophoresis analysis of PCR products amplified from genomic DNA of various bacterial pathogens. (A) PCR using the 16S-23S rRNA ITS-targeting primer set. (B) PCR using the 16S rRNA-targeting primer set. Supplementary Fig. S2. Multiple sequence alignment of the adhE-ywdF target region among fish-derived L. garvieae strains. The aligned sequences include four L. garvieae strains isolated from fish: AP027239.1, AP009333.1, AP009332.1 and AP043994.1. The target region showed 100% nucleotide identity across all tested strains, confirming that the selected target is highly conserved within L. garvieae. Supplementary Fig. S3. Sequence alignment of the target region and RPA-LFD detection of Lactococcus species. (A) Partial nucleotide sequence alignment of the adhE-ywdF target region from L. garvieae and L. petauri. The binding sites of the forward primer RPAF3, the reverse primer RPAR1-bio, and the nfo probe are indicated. (B) RPA-LFD assay results using genomic DNA from L. garvieae, L. petauri, L. lactis, and a no‑template control (NC). Positive signals (test line) were obtained for both L. garvieae and L. petauri, indicating cross‑reactivity. No amplification was observed for L. lactis or the NC. [file 12917_2026_5587_MOESM1_ESM.zip › 12917_2026_5587_MOESM8_ESM.tif]

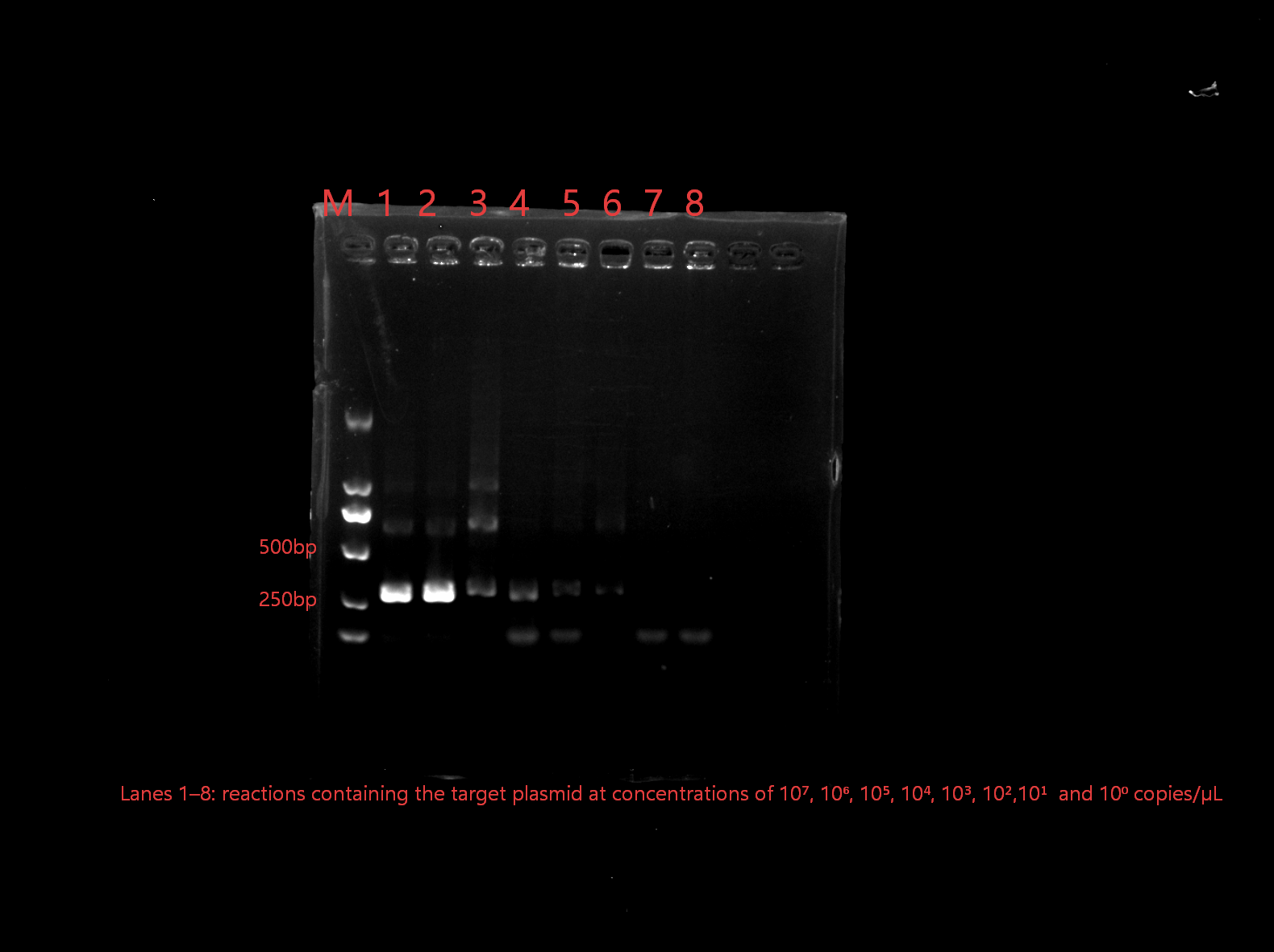

Supplement: Supplementary file 1 — Supplementary Material 1. Supplementary Fig. S1. Specificity assessment of two previously published PCR primer sets for L. garvieae detection. Agarose gel electrophoresis analysis of PCR products amplified from genomic DNA of various bacterial pathogens. (A) PCR using the 16S-23S rRNA ITS-targeting primer set. (B) PCR using the 16S rRNA-targeting primer set. Supplementary Fig. S2. Multiple sequence alignment of the adhE-ywdF target region among fish-derived L. garvieae strains. The aligned sequences include four L. garvieae strains isolated from fish: AP027239.1, AP009333.1, AP009332.1 and AP043994.1. The target region showed 100% nucleotide identity across all tested strains, confirming that the selected target is highly conserved within L. garvieae. Supplementary Fig. S3. Sequence alignment of the target region and RPA-LFD detection of Lactococcus species. (A) Partial nucleotide sequence alignment of the adhE-ywdF target region from L. garvieae and L. petauri. The binding sites of the forward primer RPAF3, the reverse primer RPAR1-bio, and the nfo probe are indicated. (B) RPA-LFD assay results using genomic DNA from L. garvieae, L. petauri, L. lactis, and a no‑template control (NC). Positive signals (test line) were obtained for both L. garvieae and L. petauri, indicating cross‑reactivity. No amplification was observed for L. lactis or the NC. [file 12917_2026_5587_MOESM1_ESM.zip › 12917_2026_5587_MOESM9_ESM.tif]
